# Blood transcriptomic signatures associated with molecular changes in the brain and clinical outcomes in Parkinson’s disease

**DOI:** 10.1038/s41467-023-39652-6

**Published:** 2023-07-05

**Authors:** Krithi Irmady, Caryn R. Hale, Rizwana Qadri, John Fak, Sitsandziwe Simelane, Thomas Carroll, Serge Przedborski, Robert B. Darnell

**Affiliations:** 1 Laboratory of Molecular Neuro-oncology, The Rockefeller University, 1230 York Avenue, New York, NY 10065 USA; 2 Bioinformatics Resource Center, The Rockefeller University, 1230 York Avenue, New York, NY 10065 USA; 3 Department of Neurology, Columbia University, 630 West 168th Street, New York, NY 10032 USA; 4 Department of Pathology & Cell Biology, Columbia University, 630 West 168th Street, New York, NY 10032 USA; 5 Department of Neuroscience, Columbia University, 630 West 168th Street, New York, NY 10032 USA; 6 Howard Hughes Medical Institute, The Rockefeller University, 1230 York Avenue, New York, NY 10065 USA

**Keywords:** Parkinson's disease, Molecular neuroscience, Parkinson's disease

## Abstract

The ability to use blood to predict the outcomes of Parkinson’s disease, including disease progression and cognitive and motor complications, would be of significant clinical value. We undertook bulk RNA sequencing from the caudate and putamen of postmortem Parkinson’s disease (*n* = 35) and control (*n* = 40) striatum, and compared molecular profiles with clinical features and bulk RNA sequencing data obtained from antemortem peripheral blood. Cognitive and motor complications of Parkinson’s disease were associated with molecular changes in the caudate (stress response) and putamen (endothelial pathways) respectively. Later and earlier-onset Parkinson’s disease were molecularly distinct, and disease duration was associated with changes in caudate (oligodendrocyte development) and putamen (cellular senescence), respectively. Transcriptome patterns in the postmortem Parkinson’s disease brain were also evident in antemortem peripheral blood, and correlated with clinical features of the disease. Together, these findings identify molecular signatures in Parkinson’s disease patients’ brain and blood of potential pathophysiologic and prognostic importance.

## Abstract (web summary)

Understanding molecular processes behind variable clinical features of Parkinson’s disease is valuable. Distinct molecular patterns in the brains, reflected in the blood, reveal mechanisms linked to clinical diversity in cognitive and motor decline.

## Introduction

Parkinson’s disease (PD) is the second most common neurodegenerative disorder after Alzheimer’s disease, affecting 2–3% of the population over 65 years of age^1–3^. Clinically, PD is a highly heterogeneous disease with motor symptoms such as tremors, slowness of movements, stiffness, and postural instability, and more than half of PD patients develop PD dementia (PDD) within 10 years of diagnosis^4,5^. Patients with late onset of PD experience faster progression and are at increased risk of dementia, while patients with early onset have slower progression, less frequent cognitive impairment, but more treatment-related motor complications^6^. The disabling motor and non-motor manifestations of PD are primarily due to the loss of dopaminergic input from the substantia nigra into the dorsal striatum^1^.

The dorsal striatum is divided into the caudate and putamen, which have discrete corticostriatal circuitry and differing roles in PD symptom manifestations^7,8^. Functional imaging studies in PD patients have demonstrated that loss of dopaminergic input into the caudate and putamen correlate with cognitive impairment and motor worsening, respectively^9–12^. While the mainstay of treatment for PD remains levodopa, which restores brain dopamine levels, its chronic use is fraught with several problems, including loss of drug efficacy, erratic (on-off) responses, and abnormal hyperkinetic movements such as levodopa-induced dyskinesia, a common motor complication related to dopamine transporter loss and abnormal connectivity in the putamen^13,14^.

While the molecular basis underlying the various clinical-pathologic features of PD remains incompletely understood, a careful assessment of molecular changes following dopamine denervation in the striatum has been limited. Two RNA sequencing (RNA-seq) studies from a small number of PD patients found downregulation of synaptic pathway RNAs in the putamen, but it is not known whether these changes are shared with the caudate or if the caudate is molecularly distinct in PD^15–18^. Moreover, changes in RNA in the caudate and putamen have not been studied with respect to the heterogeneous clinical features of PD.

Peripheral blood transcriptome studies in Alzheimer’s disease have demonstrated correlations with clinical status, including correlates of immune and neural changes with molecular brain pathology^19,20^ raising the question of whether the blood might be informative in PD. The Parkinson’s Progression Markers Initiative (PPMI), a multi-center study aggregating whole-blood transcriptome data from PD cases and matched controls in a publicly available dataset, recently identified altered expression of immunologic signatures, with an increase in neutrophil gene expression in the whole blood of patients with PD^21–23^. It is not yet known whether transcriptional changes detectable in PD blood correlate with clinical status or specific molecular changes in the brain.

Here, we performed bulk RNA-seq from paired caudate and putamen regions of 35 PD and 40 control brains to develop a comparative analysis of clinical and molecular changes in PD. We identified gene expression changes that were common to both caudate and putamen, including increased levels of RNAs encoding proteins involved in the regulation of miRNA activity and immune response, and decreased levels of RNAs encoding proteins involved in the postsynaptic membrane, synaptic signaling, mitochondrial dynamics, and lipid metabolism. We also identified regionally distinct changes that were specific to caudate and putamen that were associated with dementia and levodopa-induced dyskinesia, respectively. Later and earlier onset PD were also molecularly distinct, even at the end of their disease course. Findings in the PD brain were corroborated by analysis of bulk RNA profiles in peripheral blood obtained from the PPMI, correlating with disease severity and clinical features of the disease, and these findings were independent of medication effect. In summary, we identify molecular changes in PD caudate, putamen, and blood associated with the clinical heterogeneity in PD, providing RNA signatures of potential pathophysiologic, diagnostic, and prognostic importance.

## Results

### Transcriptome profiling identifies molecular pathways dysregulated in PD striatum

**Fig. 1:**
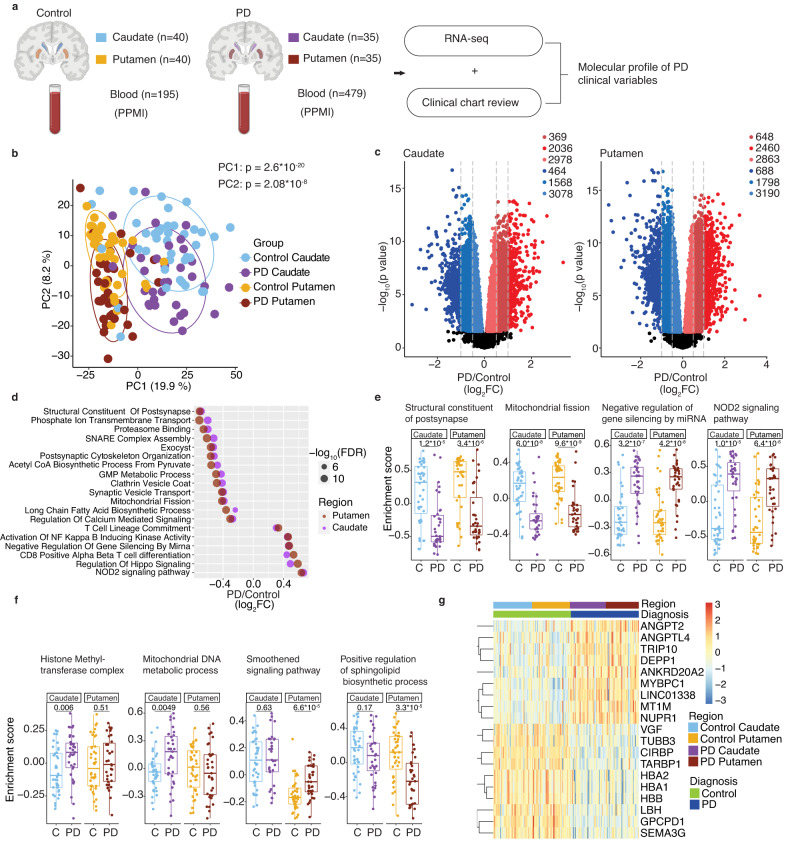
Transcriptome patterns in PD caudate and putamen. **a** Overview of the study. Bulk RNA sequencing was performed in postmortem caudate and putamen from controls and PD patients. Antemortem blood transcriptome data was obtained from an independent cohort of subjects enrolled in the PPMI study. Clinical information was extracted from accompanying charts (postmortem donors) and open-access database (PPMI subjects) to determine molecular profiles associated with PD clinical variables. Parts of the image were created with BioRender.com. **b** Principal component analysis (PCA) shows the separation of caudate and putamen regions of striatum along PC1 (*p* value: 2.6 × 10^−20^, Kruskal–Wallis test). Healthy controls and PD are separated along PC2 (*p* value: 2.08 × 10^−8^). Ellipses indicate confidence interval = 0.8 of indicated groups. **c** Volcano plot shows significantly changed RNAs in PD caudate and putamen compared to their respective controls. RNAs with significant changes in expression (determined by moderated *t*-tests with multiple test corrections using limma) with false discovery rate (FDR) <0.05 and an absolute value of log_2_ fold change (FC, PD/Control) >0.1, 0.5, and 1 are colored according to direction. **d** Bubble plot shows representative top significantly (FDR <0.05) up- (log_2_FC >0) and down (log_2_FC <0) -regulated GO terms in both caudate and putamen, as determined by GSVA. SNARE: Soluble *N*-ethylmaleimide-sensitive factor attachment proteins receptor, GMP guanosine monophosphate, NOD nucleotide-binding oligomerization domain. **e** Sample-wise gene set enrichment scores (determined by GSVA) for individual donors show select GO terms that are changed in both regions. C- Controls (*n* = 40), PD (*n* = 35). **f** Gene set enrichment scores for individual donors show select GO terms that are preferentially changed in either caudate or putamen. C- Controls (*n* = 40), PD (*n* = 35). **g** Heatmap shows expression (log_2_(counts per million), scaled by genes) of classifier genes (identified in caudate) in control and PD striatum. Significance for box plots were determined by the two-sided Wilcoxon test. Box plots show lower and upper hinges corresponding to the first and third quartiles (representing 25th and 75th percentile, respectively). Whiskers extend from the hinge to the 1.5 × inter-quartile range. The Center line indicates the median. Source data are provided as a Source Data file.

We undertook bulk RNA-seq from both the caudate and putamen from 40 control donors and 35 PD donors (Fig. 1a, and Supplementary Data S1, 2). Principal component analysis (PCA) of the top 500 variable genes revealed four significant clusters, corresponding to the striatal region and clinical diagnosis (Fig. 1b). The first principal component (PC1) was driven by the striatal region, separating caudate from putamen, while the second, PC2, was driven by disease status, separating control from PD striatum. PC2 scores correlated with tyrosine hydroxylase (TH) protein levels, thus confirming that these RNA changes reflected the extent of dopamine denervation in PD (Supplementary Fig. 1a, b and Supplementary Data S3).

Differential gene expression analysis revealed 5383 and 5110 up and downregulated RNAs, respectively, in the PD caudate compared to controls, and 5971 and 5676 up and downregulated RNAs, respectively, in PD putamen compared to controls (Fig. 1c and Supplementary Data S4,5). To identify gene sets with similar biological activity that were changed in PD, we employed gene set variation analysis (GSVA) on Gene Ontology (GO) terms^24^ (Fig. 1d and Supplementary Data S6–9). GSVA provides an enrichment analysis that takes into account all genes in a GO term/pathway using a non-parametric, unsupervised method (i.e., without relying on phenotypic/disease group information) for estimating the variation of that gene set within each sample^24^. Both caudate and putamen showed decreases in transcripts encoding proteins involved at the synapse, such as postsynaptic membrane, vesicular and ion transport, and calcium-mediated signaling (Fig. 1d, e), consistent with postsynaptic processes in response to the loss of dopaminergic input from the substantia nigra^18,25^. Other transcripts downregulated in the PD caudate and putamen encoded proteins involved in lipid metabolism and mitochondrial fission (Fig. 1d, e), both of which are implicated in nigral dopamine neuronal dysfunction and death^26,27^. Conversely, regulation of miRNA activity was highly enriched, indicating their role in the modulation of RNA levels and translation in the PD striatum (Fig. 1d, e). Pathways related to NF kappa B, a transcription factor abundantly expressed in the brain and linked to neuro-inflammation, neurogenesis, and dendrite morphogenesis, were also upregulated in PD striatum (Fig. 1d)^28^. Other immune response pathways, including those involved in T cell activity and nucleotide-binding oligomerization domain-containing protein 2 (NOD2) signaling, were highly enriched in both regions of the striatum, indicating global immune hyperactivation in the PD brain (Fig. 1d, e and Supplementary Data S8, 9).

Although the majority of differentially changed GO terms were common to both caudate and putamen, we identified a number of unanticipated region-specific RNA changes (Fig. S1c and Fig. 1f). The caudate was characterized by increased enrichment of stress response pathways, including epigenetic modifications such as histone acetylation and methylation, protein kinase C signaling, chaperone-mediated protein folding and mitochondrial DNA metabolic process (Fig. S1c and Fig. 1f). Conversely, the serotonin metabolism pathway was preferentially downregulated in the caudate (Fig. S1c), consistent with previous studies showing preferential loss of serotonin markers in the caudate more than putamen in PD^29^. Terms upregulated only in the putamen included Hedgehog signaling and connexin complex (Fig. S1c). GO terms regulating the biosynthesis of ceramides, which are substrates of the PD susceptibility gene glucocerebrosidase (*GBA)*, were preferentially downregulated in the putamen (Fig. S1c and Fig. 1f)^30^. These results suggest that while the caudate and putamen share common dysregulated pathways in PD, there are region-specific molecular changes, consistent with the unique roles different striatal regions have in clinical manifestations of PD.

Gene ontology analysis of highly changed RNAs (Supplementary Data S10), defined by using more stringent filters, identified the involvement of pathways congruent to those seen on GSVA. Downregulated terms in caudate and putamen included synaptic pathways such as trans-synaptic signaling, G protein-coupled receptor signaling, and postsynaptic membrane (Fig. S1d). Top-upregulated terms in caudate included stress response terms (cellular response to heat, chaperone-mediated protein folding), inflammatory response, and purine metabolism (Fig. S1d). In the putamen, top-upregulated terms identified ion stress response as well as inflammatory response (Fig. S1d).

To assess the value of RNA-seq in the prediction of clinical diagnosis, we undertook a machine-learning approach to identify binary classifier genes that can distinguish PD from controls. Using a group of 70% of our control and donor PD caudates as a training dataset, we discovered a set of 19 classifier genes which identified clinical diagnosis with 86% accuracy. The same set of classifier genes identified clinical diagnosis in the putamen with 90% accuracy (Fig. 1g and Supplementary Data S11).

The majority of PD donors were on dopaminergic medications. To examine whether transcriptional changes identified were impacted by medication, we tested the effect of levodopa equivalent drug dose (LEDD)^31^ in our differential gene expression model, allowing the identification of RNAs associated with PD independent of their association with drug dose. This analysis showed that >95% of RNAs were significantly up and downregulated in PD independent of drug dose (Fig. S1e), and a high correlation (*r* = 0.98, *p* < 2.2e-16) was seen for differentially changed pathways in PD before and after accounting for the effect of drug dose (Fig. S1f). These results indicate that drug dose did not make a significant detectable difference on the transcriptome in postmortem brains.

Mass spectrometry on brain samples (PD caudate (*n* = 10), PD putamen (*n* = 12), and control (*n* = 5)) confirmed a correlation between expression levels of RNA and protein in the striatum (Fig. S2a, c and Supplementary Data S12, 13). GO terms altered in the RNA-seq were similarly affected at the protein level (Fig. S2b, d and Supplementary Data S14, 15). The mitochondrial proteome was the most downregulated term in caudate (including proteins involved in the mitochondrial envelope, mitochondrial protein complex, and mitochondrial gene expression), along with ion transport and lipid metabolic processes, while immune-mediated GO terms were upregulated (Fig. S2b). In putamen, transmembrane transport, lipid metabolism, and mitochondrial envelope terms were downregulated, while inflammatory pathways were upregulated (Fig. S2d), consistent with RNA-seq. These observations provide independent support for the findings seen by RNA-seq, indicating that the RNA changes give rise to steady-state protein changes in the PD striatum.

### Concordant transcriptome patterns are seen in the PD striatum and peripheral blood

**Fig. 2:**
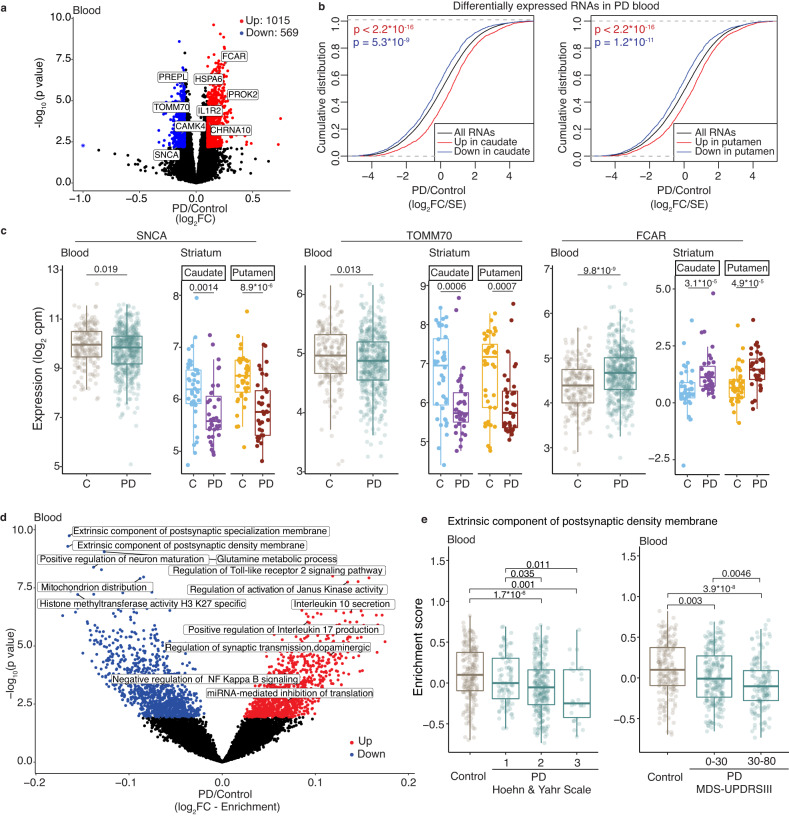
Concordant RNA changes are seen in the PD brain and blood. **a** Volcano plot showing differential gene expression analysis of blood transcriptome from PPMI subjects. Select RNAs that are also significantly up- or downregulated in the PD striatum (caudate or putamen) are labeled. Controls (*n* = 195), PD (*n* = 479). Only significantly (FDR <0.05) changed (log_2_FC >0.1 or <−0.1) RNAs in PD blood (determined by moderated t-tests with multiple test corrections using limma) are colored according to direction. Blue asterisk indicates log_2_FC < −1. **b** Cumulative distribution function plots show that top (FDR <0.05, log_2_FC >0.5, or log_2_FC <−0.5) changed RNAs in the PD caudate and putamen (*n* = 35 each) are collectively differentially expressed in the same direction in blood from controls (*n* = 195) and PD (*n* = 479) subjects in the PPMI cohort. *p* values were calculated using two-sided Kolmogorov–Smirnov tests. **c** RNA expression box plots showing log_2_(cpm) for select RNAs significantly changed in the striatum (controls (*n* = 40), PD (*n* = 35)), and blood (PPMI) in controls (*n* = 195) and PD (*n* = 479) subjects. *p* values were determined by two-sided Wilcoxon tests. C control. **d** Volcano plot shows significantly (FDR <0.05) changed GO terms in PD blood compared to controls. Select top GO terms are labeled. *p* values and FDRs are determined by moderated *t*-tests and multiple test corrections using limma. **e** Gene set enrichment scores of the top downregulated GO term (extrinsic component of postsynaptic density membrane) in PD blood correlate with disease progression, based on Hoehn and Yahr scale and MDS-UPDRS III motor scores. C-Controls (*n* = 195), PD (Hoehn and Yahr 1: *n* = 93, 2: *n* = 249, 3: *n* = 26; MDS-UPDRSIII 0-30: *n* = 224, 30–80: *n* = 144) *p* values were obtained from the Wilcoxon test. Only significant comparisons as determined by two-sided Wilcoxon tests are shown. Box plots show lower and upper hinges corresponding to the first and third quartiles (representing the 25th and 75th percentile, respectively). Whiskers extend from the hinge to the 1.5 × inter-quartile range. The center line indicates the median. Source data are provided as a Source Data file.

Peripheral blood transcriptome studies in Alzheimer’s disease have reported findings that mirror brain pathology^19,20^. Therefore, we tested whether the transcriptional changes we identified in PD caudate and putamen were also altered in antemortem blood collected from PD patients (*n* = 479) compared to control donors (*n* = 195) in the PPMI cohort (Supplementary Data S16). About 1015 and 569 RNAs were significantly up and downregulated in PD blood (Fig. 2a and Supplementary Data S17), consistent with the prior analysis of this data^23^. We observed that RNAs significantly up or downregulated in the PD caudate and putamen were altered in the same direction in the blood (Fig. 2b).

To confirm this observation, we analyzed blood bulk RNA-seq data from an independent cohort, the Parkinson’s Disease Biomarker Program (PDBP). There was a significant correlation between differentially expressed PD RNAs detected in the PPMI and PDBP cohorts (Fig. S3a). Cumulative changes in RNAs significantly up or downregulated in the PD brain were changed in the same direction in the PDBP blood (Fig. S3b and Supplementary Data S18). While the evaluation of a large number (>5000, Fig. S4a) of significant striatal PD-associated RNAs in the blood expectedly limit the overall effect size in blood, these findings indicate consistent concordance between postmortem brain and antemortem blood RNAs in PD.

For example, *SNCA*, which encodes alpha-synuclein and is linked to a familial form of PD^32^, was significantly downregulated in both the blood and brain, consistent with smaller targeted RNA studies in the blood and with prior analysis of PPMI data (Fig. 2a, c)^23,33^. *TOMM70*, an import receptor involved in the import of *PINK-1* into the mitochondria^34^, was also significantly downregulated in PD caudate, putamen, and blood (Fig. 2a, c) as was the calcium-dependent kinase *CAMK4* (Fig. 2a). Upregulated RNAs discovered in both the blood and the brain were immune pathway genes including *FCAR* (Fig. 2a, c) and *IL1R2*, stress-induced molecular chaperone *HSPA6* and genes important in brain function such as the G protein-coupled receptor *PROK2* and neuronal acetyl cholinergic receptor *CHRNA10* (Fig. 2a).

Significantly changed GO terms detected in the PD caudate and putamen were also collectively consistent in their direction of change in PD peripheral blood (Fig. S4b and Supplementary Data S19). Similar to our observations of RNA changes in PD caudate and putamen, transcripts encoding proteins in miRNA regulation and immune response (Toll-like receptor signaling, interleukin secretion, and NF kappa B signaling) were upregulated in the blood (Fig. 2d). Conversely, again consistent with observations in the PD caudate and putamen, where we observed downregulation of neuronal and synaptic pathways, GO terms downregulated in PD blood included postsynaptic membrane, glutamine metabolic process and neuron maturation (Fig. 2d). Direction of change in top differentially changed GO terms in PD blood were driven by changes in most genes in the GO term (Fig. S4c).

The top GO terms identified as dysregulated in GSVA of PD blood were neuronal, such as “extrinsic component of postsynaptic specialization/density membrane”, and inversely correlated with motor severity, based on two different clinical scales (the Movement disorders society (MDS)-unified Parkinson’s disease rating score (UPDRS) and the modified Hoehn and Yahr scale)^35,36^ (Fig. 2e). Similarly, *TIAM-1*, a gene in the postsynaptic membrane GO term that encodes a known modulator of glutamatergic synapse function^37,38^, was significantly decreased in PD caudate, putamen, and blood, and its expression inversely correlated with motor severity in the PPMI cohort (Fig. S4d). In summary, peripheral blood from antemortem PD subjects shows concordant changes in RNA expression with PD brain, and strikingly, these antemortem blood signatures correlate with PD progression.

We also observed concordance in expression patterns between the blood of prodromal subjects^23^ (*n* = 60) who were not clinically diagnosed with PD, but had clinical signs suggesting a risk for PD (such as anosmia, sleep disorder, and dopamine transporter scan showing loss of dopamine), and postmortem PD caudate (Fig. S4e). In contrast, compared to controls, carriers of PD-related genetic mutations who were otherwise healthy, did not show concordance with the direction of change in PD brain RNA (Fig. S4e and Supplementary Data S20), suggesting that such concordance is detectable in the blood when pathology has also manifested in the brain.

We next investigated if transcriptome changes seen in PD blood were influenced by medication use. In PD blood, ~40% of RNAs changed independent of medication dose (LEDD) (Fig. S5a).

A small number of blood RNAs (38) correlated with LEDD (Supplementary Data 21). Overall, the concordance between the blood and brain transcriptome remained true with differentially expressed RNAs and pathways independent of medication dose (Fig. S5b, c).

To further investigate the concordance between blood and brain in PD, we analyzed blood transcriptome changes in PD patients when they were drug-naive for dopaminergic medications. As expected, drug-naive PD subjects had lower MDS-UPDRS scores compared to those on medications (*p* value <2.2e-16) (Fig. S5d). While this makes a complete uncoupling of the effect of medication and disease challenging, we nonetheless compared the transcriptome profile of drug-naive PD subjects to controls. This revealed that 34 and 17% of up- and downregulated RNAs in drug-naive PD blood were concordant with those seen in the PD striatum (Fig. S5e and Supplementary Data S22). For example, *SNCA* and *FCAR* were significantly down- and upregulated in PD blood, even in drug-naive PD subjects (Fig. S5f, g). Similarly, RNAs downregulated in the PD brain, such as the neuronal enriched RNA *GPR52* and the PD gene *DNAJC6*, were also downregulated in PD blood in drug-naive patients (Fig. S5f, g). On GSVA, differentially changed pathways showed a significant correlation between drug-naive and PD subjects on dopaminergic medications (Fig. S5h and Supplementary Data S23).

These observations were supported with data from an independent analysis that used machine learning to determine RNAs that serve as classifiers in the blood to distinguish controls and PD among early-stage (average disease duration of 2 years) drug-naive PPMI subjects^39^. We observed that up- and downregulated classifier RNAs in the blood from early-stage drug-naive PPMI subjects were collectively changed in the same direction in the postmortem striatum, indicating that the PD blood transcriptome reflects changes in the PD brain independent of medication status (Fig. S5i).

### Distinct transcriptome profiles are associated with cognitive and motor complications of PD

**Fig. 3:**
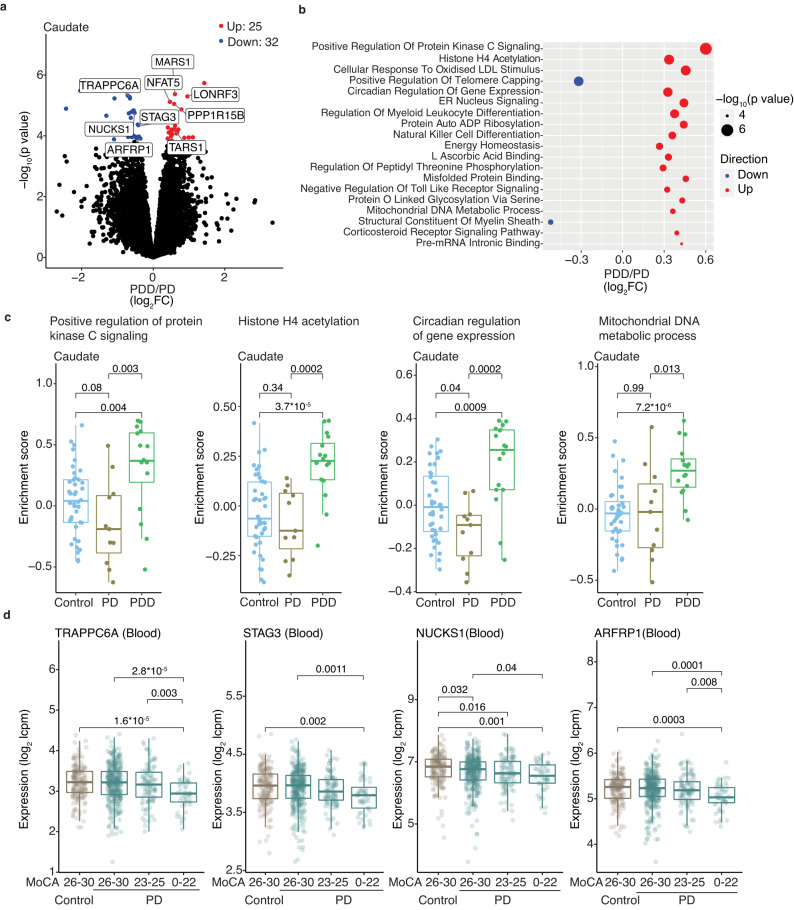
Caudate is affected in PDD with concordant changes seen in blood. **a** Volcano plot shows differentially expressed RNAs in PDD caudate (*n* = 16) compared to PD without dementia (PD) (*n* = 11). Only significantly (FDR <0.05) changed (absolute value of log_2_FC >0.1) RNAs (determined by moderated *t*-tests with multiple test corrections using limma) are colored according to direction. **b** Bubble plot shows representative top significant (FDR <0.05) GO terms from GSVA analysis in PDD caudate compared to PD. *p* values were determined by fgsea^75^, with multiple test correction using the Benjamini–Hochberg method. LDL low-density lipoprotein, ER endoplasmic reticulum, ADP adenosine diphosphate. **c** GSVA enrichment scores of select GO terms in controls, PD, and PDD. Controls (*n* = 40), PD (*n* = 11), PDD (*n* = 16). *p* values were determined by two-sided Wilcoxon tests. **d** Differentially changed RNAs in the PDD caudate show similar changes in expression levels in PD blood with worsening cognition as seen by Montreal Cognitive Assessment (MoCA) scores. Controls (*n* = 161), PD: MoCA 26-30 (*n* = 332), 23–25 (*n* = 96), 0–22 (*n* = 48). Only significant comparisons as determined by two-sided Wilcoxon tests are shown. Box plots show lower and upper hinges corresponding to the first and third quartiles (representing the 25th and 75th percentile, respectively). Whiskers extend from the hinge to the 1.5 × inter-quartile range. The center line indicates the median. Source data are provided as a Source Data file.

As pathology in the caudate, but not putamen, is associated with Parkinson’s disease dementia (PDD)^9–12,40–42^, we next assessed whether RNAs changed in PDD did so in a region-specific manner. Sixteen donors had a diagnosis of PDD and 11 had PD without dementia (see Methods, Supplementary Data S24). Differential gene expression analysis in caudate identified 57 RNAs that were significantly changed in PDD compared to PD without dementia, while only eight RNAs were significantly affected in the putamen (Fig. 3a, Fig. S6a, and Supplementary Data S25, 26).

GSVA identified more than 300 GO terms that were significantly changed in PDD compared to PD caudate (Fig. 3b, Fig. S6b, and Supplementary Data S27, 28). No significant GO terms were identified in the putamen in the same patients, suggesting that the region-specific changes seen in the caudate are specifically linked to dementia (Fig. S6b). Significant GO terms in PDD caudate included circadian regulation of gene expression and stress response pathways, including protein kinase C signaling, histone H4 acetylation, and mitochondrial DNA metabolic process (Fig. 3b, c).

We next assessed the blood transcriptome in PPMI PD patients with respect to clinical measures of cognitive impairment, using the MoCA (Montreal Cognitive Assessment) scale where MoCA ≥26 is normal; *n* = 144) and MoCa <26 is abnormal; *n* = 332) (Fig. S6c and Supplementary Data S29, 30). RNAs that were differentially expressed in the caudate in PDD patients, such as *TRAPPC6A*, *ARFRP1, NUCKS1*, and *STAG3*, were also changed in the blood with worsening MoCA (Fig. 3d).

The top GO terms that differentiated subjects with and without cognitive impairment in the blood were neuronal terms, including glutamatergic signaling (glutamate binding, NMDA glutamate receptor complex, spontaneous neurotransmitter secretion) (Fig. S6d and Supplementary Data S31). GO terms dysregulated in PDD caudate such as histone H4 acetylation, mitochondrial DNA metabolic process, and circadian regulation of gene expression were inversely changed in the blood of PPMI subjects with cognitive impairment (Fig. S6d, e). As postmortem PDD donors had longer average disease duration (13.4 ± 7.2 years) relative to PPMI PD subjects with cognitive impairment (average disease duration 6.7 ± 6.3 years), these observations suggest that the above stress response pathways are important in the development of PDD, and likely undergo dynamic changes early in the course of the disease.

While PPMI PD subjects, when drug-naive for dementia medications, expectedly had significantly better cognitive profiles (Fig. S6f), many of these RNAs and pathways nonetheless showed similar expression patterns with decreasing MoCA scores (Fig. S6g, h). Together, these observations indicate that RNAs associated with dementia in the brain show corresponding changes in the blood of PD subjects with worsening cognition.

We next assessed the molecular changes in caudate and putamen in PD subjects with a clinical history of a motor complication-levodopa-induced dyskinesia (Supplementary Data S32), although only small numbers of comparable donors with (*n* = 5) or without (*n* = 11) dyskinesia were available for analysis. There was no significant difference in the average LEDD between donors with (average LEDD 716.3 ± 577.5 mg) and without dyskinesia (average LEDD 954.7 ± 780.1 mg). Differential gene expression analysis identified 18 RNAs which were altered in the putamen, while few RNAs were differentially expressed in the caudate (Fig. S7a and Supplementary Data S33, 34). We assessed transcriptome patterns in the blood of PPMI subjects with (*n* = 213) and without (*n* = 201) history of dyskinesia, but did not observe any significant difference between these groups (Supplementary Data S35). In contrast to brains from donors with PDD, where differentially changed GO terms were observed in the caudate, brains from donors with levodopa-induced dyskinesia showed differentially changed GO terms specifically in the putamen (Fig. S7b and Supplementary Data S36,37). Here, we identified differences in the regulation of endothelial cell pathology and cell death GO terms (endothelial cell apoptosis, anoikis, vascular permeability, fluid laminar shear stress, death receptor signaling; Fig. S7c, d). These observations were consistent with previous functional imaging studies in PD patients and animal models of PD, showing vascular pathology in the putamen in levodopa-induced dyskinesia^43–46^. Taken together, our results suggest that PD patients with cognitive and motor complications have distinct molecular changes in the caudate and putamen respectively, some of which are reflected in the antemortem blood transcriptome.

### Differential molecular patterns in the striatum and blood reflect age at the onset of PD

Patients with late-onset PD are clinically distinct from patients with early-onset of disease, with late-onset PD patients experiencing faster motor and non-motor progression of disease compared to early-onset PD patients^47–49^. While the classic definition of early onset is arbitrary, with ages ranging from <40 to <50^50,51^, previous studies have shown that for every decade for onset studied, older age of onset was associated with worse motor and non-motor disease^48^. This led us to hypothesize that there may be distinct molecular features in the striatum that distinguish the age of PD onset.

**Fig. 4:**
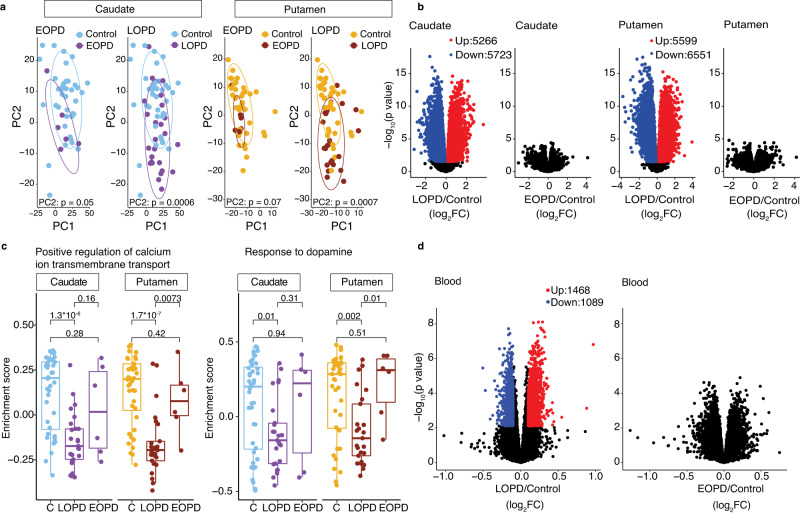
PD caudate and putamen from patients with later onset PD (LOPD) show significant changes in RNA levels compared to controls. **a** PC2 values (from Fig. 1b) distinguish LOPD (*n* = 25), but not EOPD (*n* = 6) samples from controls (*n* = 40) in both the caudate and putamen. For each region, controls are plotted individually with EOPD (left) and LOPD (right) samples and colored accordingly. *p* values for separation of the groups along PC2 were obtained from Kruskal–Wallis test. *p* = 0.05 (Caudate, EOPD and Control), *p* = 0.0006 (Caudate, LOPD and Control), *p* = 0.07 (Putamen, EOPD and Control), *p* = 0.0007 (Putamen, LOPD and Control). Ellipses indicate confidence interval = 0.8 of indicated groups. **b** Volcano plots show differential gene expression for LOPD and EOPD compared to controls in caudate (left) and putamen (right). Only significantly changed RNAs (FDR <0.05, absolute value of log_2_FC >0.1) are colored according to direction. p values are determined by moderated *t*-tests with limma and FDRs are determined by multiple test corrections using the Benjamini–Hochberg method. **c** Gene set enrichment scores for GO terms are plotted for LOPD, EOPD, and controls (C) in the caudate and putamen. Changes detected in PD donors with LOPD when compared to controls, are not significant in those with EOPD compared to controls. *p* values are the result of the two-sided Wilcoxon test. Box plots show lower and upper hinges corresponding to the first and third quartiles (representing the 25th and 75th percentile, respectively). Whiskers extend from the hinge to the 1.5 × inter-quartile range. The center line indicates the median. **d** Volcano plots show differential gene expression analysis in LOPD and EOPD compared to controls in the blood. Only RNAs with FDR <0.05 and an absolute value of log_2_ FC >0.1 are colored according to direction. Control (*n* = 195); LOPD (*n* = 330); EOPD (*n* = 149). Source data are provided as a Source Data file.

Indeed, PCA revealed that patients with later age of onset (referred to as LOPD, age of onset > 55) separated better from controls, more than patients (*n* = 6) with earlier onset of PD (referred here as EOPD, age of onset ≤55) (Fig. 4a and Supplementary Data S38). After factoring in age at death, differential gene expression analysis in the caudate of LOPD patients identified 5266 and 5723 RNAs that were significantly up or downregulated, respectively (Fig. 4b and Supplementary Data S39). In the putamen of LOPD patients, 5599 and 6551 RNAs were up or downregulated, respectively (Fig. 4b and Supplementary Data S40). LOPD brains showed differentially expressed RNAs compared to controls even when small sample numbers were used (*n* = 6), again suggesting that changes in the LOPD striatum are robust (Fig. S8a, b). Few differentially expressed RNAs could be identified between EOPD patients and controls (Fig. 4b, Fig. S8b, and Supplementary Data S41, 42).

Calcium ion transport was the top changed GO term in LOPD, consistent with significant synaptic dysregulation seen in PD. GSVA showed significant downregulation of this pathway in LOPD, while EOPD patients showed modest differences relative to controls (Fig. 4c and Supplementary Data S43, 44). A more targeted approach, looking at dopaminergic pathway differences, showed the same differences in molecular impact in LOPD but not EOPD (Fig. 4c). Notably, these differences were seen despite the shorter disease duration in LOPD (11.24 ± 5.9 years) compared to EOPD (20.50 ± 5.1 years).

Functional imaging studies in PPMI subjects have demonstrated that dopamine dysfunction is comparable in the putamen in late and early-onset PD^48^. Postmortem studies have suggested that there is a substantial loss of DA input into the putamen within four years of disease onset, although this has not been investigated in the context of the age of onset of disease^52^. Given the modest molecular changes in EOPD, we tested the hypothesis that this was secondary to preserved dopamine input in these patients. We assessed TH levels on protein extracts from caudate and putamen of donors with LOPD and EOPD to estimate the loss of dopaminergic innervation in these groups. Both LOPD and EOPD showed significant loss of TH protein (Fig. S8c–e). These observations show that despite the comparable loss of nigral dopamine input and shorter disease duration compared to EOPD, LOPD patients show significant RNA changes. Taken together, these data suggest physiologic differences in how the striatum responds to dopamine loss in LOPD and EOPD.

We next analyzed the blood transcriptome from controls (*n* = 195), LOPD (*n* = 330), and EOPD (*n* = 149) individuals from the PPMI cohort (Supplementary Data S45). Similar to our observations in the caudate and putamen, significant blood RNA changes were evident in LOPD but not EOPD (Fig. 4d and Supplementary Data S46, 47) when compared to controls. These observations suggest that LOPD and EOPD are fundamentally different in their molecular characteristics in the caudate and putamen, and these differences are mirrored in the blood.

### Temporal dynamics of molecular pathways in PD striatum

**Fig. 5:**
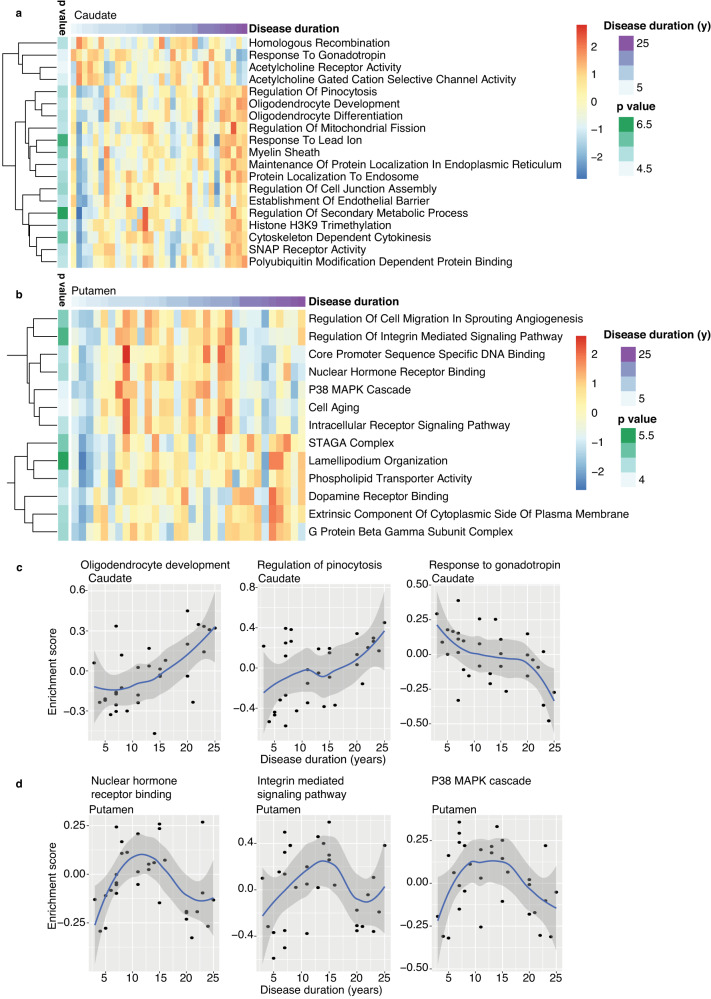
Disease duration correlates with distinct molecular changes in caudate and putamen. **a**, **b** Heatmaps show significant (FDR <0.05) GO terms associated with disease duration in PD caudate (**a**) and putamen (**b**), identified by spline regression analysis. Scaled expression (log_2_(cpm)) values are plotted. y years. **c**, **d** Scatter plots show GSVA enrichment scores for top significant GO terms plotted against disease duration in caudate (**c**) and putamen (**d**). Blue lines indicate loess regression lines, with 95% confidence intervals shaded in gray. Source data are provided as a Source Data file.

Previous studies have shown an association between disease duration and loss of dopamine input into the striatum^52^. This association was non-linear, with the greatest loss of dopamine input into the putamen occurring in the first four years after diagnosis. We used spline interpolation in the brain to investigate relationships between RNA levels and disease duration in the striatum. GO terms significantly associated with disease duration in the caudate included oligodendrocyte development, pinocytosis, and gonadotropin response pathway, the latter being consistent with extra-hypothalamic gonadotropin expressing population discovered in the striatum^53^ (Fig. 5a, c and Supplementary Data S48). In the putamen, GO terms associated with increased disease duration include nuclear hormone receptor binding, integrin signaling, and cellular senescence (p38 Mitogen-activated protein (MAP) kinase, cell aging) (Fig. 5b, d and Supplementary Data S49). In PD putamen, many pathways showed downregulation with long (≥20 years) disease duration, which may reflect the dynamic changes that occur in the final stages of the disease. A similar analysis in peripheral blood from PPMI subjects was precluded by a narrow range and relatively short disease duration in this cohort (Fig. S9). In sum, these observations highlight the temporal dynamics of molecular pathways and the continuous accumulation of pathology in PD caudate and putamen over the course of the disease, from onset to death.

## Discussion

Loss of dopamine input into the striatum is one of the key mechanisms underlying clinical manifestations in PD. At the time of diagnosis of PD, most patients have lost roughly 60% of the nigral dopamine neurons^3^. Although the initial loss of dopamine input is balanced by compensatory mechanisms in the striatum, eventually, striatal dysfunction leads to the onset of symptoms^54^. Therefore, understanding the molecular changes in the striatum in PD has a vast potential for disease modulation. Moreover, the biological and molecular basis for clinical heterogeneity seen in PD is not understood. Identifying molecular features in the blood that correlate with pathological changes in the brain and clinical trajectories offers predictive value for patients early in the course of the disease. Here, we present an in-depth molecular analysis of the PD striatum and correlate molecular changes in the striatum with clinical variables. We discovered that the caudate and putamen show both overlapping and distinct transcriptome changes, with the caudate particularly affected in PD patients with cognitive impairment, while the putamen is more affected in motor complications such as dyskinesia. We show that LOPD is molecularly distinct from EOPD, even at the end of their disease course. Finally, we identify that molecular signatures of PD and its clinical heterogeneity can be identified in the peripheral blood antemortem, which could ultimately be clinically useful for prognosis and treatment.

We observed that several GO terms previously linked to the death of substantia nigra dopaminergic neurons, including immune response, and mitochondrial dynamics, were also altered in the striatum, thus indicating a global dysregulation of these pathways in PD^1^. In PDD caudate, we observed an upregulation of stress response pathways linked to memory formation in mice, such as histone acetylation and protein kinase C signaling^55–57^ and mitochondrial DNA metabolism, consistent with a previous study in the PDD prefrontal cortex^58^. Functional imaging studies have shown abnormal putaminal neurovascular responses in patients with levodopa-induced dyskinesia^46^, linked to increased endothelial proliferation, neo-angiogenesis, and blood–brain barrier permeability^43–45^. Our discovery of dysregulated vascular endothelial pathways provides molecular confirmation of these mechanisms in PD patients with levodopa-induced dyskinesia.

Our observations of more robust molecular changes in LOPD than EOPD, despite their longer disease duration and comparable loss of dopaminergic innervation, may explain the faster disease progression in late-onset PD patients^47^ and indicate different pathophysiologies and response to dopamine loss in the two groups. As RNA changes associated with the age of death in the two groups were factored in our comparative analysis, it is likely that there is an additive effect of aging on disease progression, reflected in the more severe molecular changes seen in LOPD. Our findings establish a molecular basis for clinical differences with age at onset in PD and should be considered in the recruitment of subjects to clinical trials, to minimize heterogeneity and improve the efficacy of novel treatment measures.

A previous study that combined transcriptomics in PD substantia nigra with a mouse cell-type specific transcriptome atlas has identified that oligodendrocyte genes are significantly upregulated in PD, even in early preclinical stages^59^. Our observation that RNAs involved in oligodendrocyte development are altered with disease duration indicates that these cells are additionally involved in PD striatum. Nuclear hormone receptor signaling, associated with disease duration in the putamen, has been shown to be crucial for the development and physiology of the striatal dopaminergic system^60,61^. In the PD putamen, cellular senescence, including cell aging and p38 kinase cascade GO terms were also associated with disease duration. Cellular senescence, once thought to be limited to proliferating cells, have lately been also identified as a feature of aging post-mitotic neurons and in nigral astrocytes in PD^62–64^. Activation of p38 MAP kinase is detrimental to neurons in Alzheimer’s disease and Huntington’s disease^65,66^. Clearance of senescent cells have also been shown to mitigate neurodegeneration^67,68^. These findings invite considerations for investigations into senolytics currently in clinical trials, for PD^69^.

Our observations in the blood transcriptome of PPMI subjects further supports and extends our observations of striatal transcriptome changes seen in PD. Consistent with previous analysis of blood transcriptomes in PD, we noted increased immune activation in the striatum and peripheral blood^23^. Detection of RNA changes in the brain and blood may, in part, support the hypothesis that PD is a multi-system disorder with global molecular dysfunctions. As RNAs encoding proteins involved in neuronal functions distinguish controls from PD and also correlate with motor progression and disease severity, some of the peripheral changes detected in the blood may also be secondary to changes in the brain. A recent study has reported that brain genes defined in single cell-atlas are detectable in cell-free RNA from the blood^70^. A study of cell-free RNA profiles in Alzheimer’s disease blood have reported the detection of brain RNAs resembling the dysregulation seen in postmortem brains, and disease-relevant transcripts in the blood correlated with the severity of dementia^20^. A clear mechanism for how brain transcripts could gain access to the blood or how peripheral blood mirrors changes in the brain in the course of neurodegeneration is lacking, and further investigation is required to confirm these suggestions. Nevertheless, coordinated transcriptome changes seen in PD striatum and blood that correlate with heterogeneous features of the disease, are of clinical value. Molecular changes occurring in the PD brain cannot be examined non-invasively antemortem, limiting our understanding of the cause, progression, and heterogeneity of the disease. The concordant transcriptome changes seen in the brain and blood can be leveraged to molecularly characterize clinical subtypes of PD and develop novel tools for antemortem clinical diagnosis, prognostication, and monitoring therapeutic responses by a minimally invasive approach.

## Methods

### Ethical considerations

The research conducted is in accordance with ethical guidelines and regulations. The use of postmortem brain samples obtained from brain banks was governed by a Material Transfer Agreement, which ensured that the samples were obtained and used in accordance with legal and ethical requirements. The brain banks followed appropriate consent procedures from donors or their legal representatives for the collection and distribution of postmortem brain tissue for research use. This study also incorporated publicly accessible blood transcriptome data from de-identified individuals who provided informed consent at data collection sites^23^. The utilization of these data followed the guidelines outlined by the data repository’s Data Use Access policies. The research outline and protocol, including the use of postmortem brain samples and publicly available blood transcriptome data, were reviewed by the Chair of Rockefeller University’s Institutional Review Board (IRB).

### Human brain tissue

Brain tissue from individuals who had pathological confirmation of PD were obtained from NIH NeuroBioBank. Control donors did not carry a clinical history of neurological disease. Chart review of available clinical material for information on age of onset/diagnosis, disease duration, medications, dementia, and dyskinesia were done retrospectively from visit notes with movement disorders specialists, neurologists, and general and other specialty practitioners as available. Tissue banks used co-ordinates from two previously published reference atlases for dissecting caudate and putamen, including Ding et al., Figure 21 (page 42) and Figure 22 (page 44)^71^ and from Atlas by Roberts et al., map levels 16–18 (caudate) and 19–21 (putamen)^72^. Details for the categorization of individual clinical features are described in the bioinformatics section of methods. LEDD was calculated from all listed dopaminergic medications^31^.

### RNA-seq

Bulk RNA from brain tissue was extracted using the standard TRIzol (Invitrogen) extraction method. RNA samples were treated with RQ1 RNase-free DNase (Promega), purified by TRIzol LS (Ambion), and checked on Agilent Bioanalyzer. The sequencing libraries were prepared for polyadenylated RNA by using Dynabeads RNA Purification Kit (Thermo Fisher Scientific) and TruSeq Stranded Total RNA Gold Library Kit (Illumina) following the manufacturer’s instructions. IDT for Illumina TruSeq UD Indexes were used and high-throughput sequencing was performed on Illumina NovaSeq 500 to obtain 150 nucleotide paired-end reads.

### Mass spectrometry

Approximately 35 mg brain tissue was homogenized in an 8 M urea lysis buffer (8 M urea, 100 mM Na2HPO4, pH 8.5) with HALT protease and phosphatase inhibitor cocktail (Thermo Fisher). Following sonication and centrifugation, protein concentration and integrity of the supernatant was tested by bicinchoninic acid (BCA) assay (Pierce) and gel electrophoresis, respectively. Disulfide bonds were reduced with dithiothreitol and cysteines were alkylated with iodoacetamide. Proteins were extracted using Chloroform/Water/Methanol precipitation and digested using LysC (Thermo Fisher) and trypsin. Peptides were labeled with tandem mass tag (TMT) pro labeling reagent and stoichiometry along with labeling efficiency was evaluated. TMT-peptides were mixed according to label check and purified using reverse phase (RP) solid phase extraction. Purified TMT-peptides were fractionated first using strong cation exchange, and then by high-pH RP-fractionation. The resulting fractions were analyzed by liquid chromatography with tandem mass spectrometry. Spectra were queried against the *homo sapiens* proteome concatenated with common contaminants at 1% FDR. Quantitation was performed using reporter ions from fragment spectra, requiring a spectral purity of 75%. Quantitative values for proteins were log_2_ transformed. It was required that a protein be observed in at least five replicates in at least one group. The protein isoform with the highest expression was used. After this filtering, 6994 (caudate) and 5183 (putamen) proteins remained. Missing values were imputed with random low-abundant values from a normal distribution. Values were normalized to the median for each channel by subtraction. Significance was tested using permutation-based FDR-corrected *t*-tests (FDR 0.05).

### ELISA

Tissue was homogenized in Radio-immunoprecipitation (RIPA) Buffer (Sigma-Aldrich R0278) with a complete Mini EDTA-free Protease Inhibitor (Roche). The protein concentration of the lysate was quantified by a BCA protein assay kit (Pierce). About 120–200 micrograms of total protein from the striatum were used for quantification of Tyrosine hydroxylase using a sandwich ELISA kit (Antibodies online-ABIN6960326) according to the manufacturer’s instructions.

### Western blotting

Protein lysates were run on NuPAGE gels (Invitrogen) and transferred to a polyvinylidene fluoride membrane. The protein of interest was detected using ECL Prime Western Blotting Detection Reagent (Amersham - RPN2232) with an appropriate conjugated secondary horse radish peroxidase (1:15000) antibody. Antibodies used were: Tyrosine hydroxylase (Abcam AB112: 1:1000) and GAPDH (AM4300 Invitrogen 1:30000). Full scan blots are shown as source data file in the supplementary figures.

### Bioinformatics

#### General

Transcript expression was quantified from RNA-seq reads using salmon (version 0.8.1) and hg38 UCSC knownGene gene models (TxDb.Hsapiens.UCSC.hg38.knownGene, version 3.4.0). To account for differences in RNA integrity number (RIN), differential gene expression analysis was done with limma^73^ using the voomWithQualityWeights function in order to down weight low-quality samples^74^. GSEA analysis was performed using the fgsea R package^75^ (https://www.biorxiv.org/content/10.1101/060012v3) using C5 gene ontology sets (https://www.gsea-msigdb.org), and Gene set variation analysis was performed using GSVA^24^. Image visualization was done by ggplot2 (v3.5.0).

#### Principal component analysis (PCA)

For PCA analysis, expression values from voomWithQualityWeights were corrected with the removeBatchEffect function in limma to remove effects: gender and quality (using RIN values). The top 500 variable RNAs were used for PCA analysis. Kruskal–Wallis tests were performed using the correlatePCs function from the pcaExplorer package in order to test for significant correlations between PCs and experimental variables.

#### Differential gene and pathway expression—PD vs Control (brain)

Genes were filtered using the filterByExpr function from the edgeR package using default settings^76^. Samples were grouped according to brain region and disease state (PD or Control). In order to account for differences in death age in PD samples, death age was included as a covariate in the limma model. “0 + group + death age” was used as the design. voomWithQualityWeights from limma was used for normalization in order to account for sample quality. A linear model was fitted with the lmFit function, followed by computation of log_2_FC and moderated t-statistics with the eBayes function. In contrast, PD - Control was evaluated for each brain region. Multiple hypothesis testing was performed with the decideTests function, using the Benjamini–Hochberg (“BH”) method. Significant RNAs were defined as those with an FDR <0.05 and log_2_FC >[0.1] or <[−0.1]. To test for LEDD-independent transcriptional changes in the brain, Control LEDD values were set to 0, and LEDD was added to the model as a continuous variable (“0 + group + death age + LEDD”).

#### Gene set enrichment analysis—Mass Spectrometry

GSEA was performed in order to determine GO pathways enriched in mass spectrometry data for comparing PD and control (Fig. S1a). To do this, mean expression values were determined for PD and Control, and the log_2_ fold change values (PD/Control) were calculated from these values. For GSEA, genes were ranked according to log_2_ fold changes.

#### Gene set variation analysis

For all GSVA analyses, count data were processed as described for limma and log_2_-cpm values were obtained using the cpm function from edgeR and a prior count of 3. GSVA scores were calculated for all GO pathways with ten or more genes in the brain or five or more genes in the blood with the gsva function from the GSVA package, followed by the addition of sample weights with the arrayWeights function from the limma package. The resulting scores were used for standard differential expression testing with limma as described above using the same model and contrast as described for limma. The GSVA scores (which were calculated for all samples) were also used for plotting and *t*-tests among clinical subtypes using the compare_means function in the ggpubr package.

#### Gene ontology

Enrichment of GO terms was performed by the goseq package in R (v1.46.0)^77^. All expressed transcripts were used as background, and gene sets were determined using log2FC and/or FDR cutoffs as described in individual figure legends. Multiple correction was performed using the p.adjust function using the “BH” method.

#### Classifier genes

To identify classifier genes for PD and control patients, we used the MLSeq Bioconductor package (version 2.12.0)^78^ to implement a nearest shrunken centroid classifier on the voom variance stabilized caudate RNA-seq data. We used 70% of all samples as our training set and 30% as our test dataset. The accuracy of models on test data were recorded. Following this, the accuracy of the caudate classifier was also assessed in the putamen. Statistics was derived from McNemar’s test in the R stats package using default parameters.

#### Blood RNA-seq

PPMI whole blood transcriptome and clinical data were collected according to the PPMI study protocol (www.ppmi-info.org/access-dataspecimens/download-data). For up-to-date information on the study, visit ppmi-info.org. Transcript abundance files generated by salmon were obtained from the PPMI database. The tximport package was used to generate a count matrix^79^. The counts matrix filtered for the desired samples was used as input for limma and voomWithQualityweights, as described for brain samples above. For the PDPB validation cohort, the counts matrix, which contains reads mapped with subread and quantitated with featureCounts, was obtained from the Accelerating Medicine Partnership® Parkinson’s Disease (AMP PD) consortium.

#### Differential gene expression—PD vs control (blood)

About 195 healthy controls with no neurological disease and 479 subjects with a primary diagnosis of PD (including 61 Scans Without Evidence of Dopaminergic Deficit at the time of inclusion) were included in our PD versus Control analysis. For each patient included in the study, sequencing and clinical information associated with only the latest study visit was used for analysis. RNA quality (defined by the percent of reads mapping to the genome), gender, and age were included in the limma model to control for these variables. Age was calculated by using age at enrollment and visit year at sample collection. GSVA analysis was performed as described above. A sequencing plate was used to determine the batch. To test for LEDD-independent transcriptome changes, LEDD was also included in the limma model. For validation with the PDBP cohort, the most recent sample was chosen for each of a random sampling of 200 patients. As described previously^23^, limma model included sex, plate (to remove batch effects), age, and quality metrics - useableBases and strandBalance. Only protein-coding genes were included in the analysis. To test for reproducibility, six random sample sets were obtained, and representative data were shown in Supplementary Fig. 3.

#### Differential gene expression—genetic carriers vs Control (blood)

RNA-seq data of patients with PD-relevant genetic mutations but no disease were obtained from the PPMI cohort (“Genetic unaffected”, 247 patients) from AMP PD. Only those samples where age, quality, gender, and batch information were available were included in the analysis. Most recent samples were used for analysis and compared to healthy controls (183).

#### Differential gene expression—PD vs control (blood and drug-naive cohort)

To determine PD-dependent changes in dopaminergic drug-naive PD patients, the most recent RNA-seq samples when patients were drug-naive were taken into analysis. Sixteen subjects in the cohort whose chart did not indicate that they were drug-naive in the PPMI study were removed from the analysis. For limma, gender, age, quality, and batch were included in the model.

#### Differential gene expression—dementia (brain)

PD caudate (*n* = 35) samples were further categorized as PD dementia (PDD), PD without dementia (PD), according to clinical records. For the selection of PDD and PD without dementia, a chart history of dementia (“+”) was supported by medication history for cholinesterase inhibitors or NMDA receptor antagonists for dementia or scores from a cognitive test (Supplementary Data S24). In those considered not to have dementia (“−”) chart reviews did not mention dementia or a medication history for dementia. One donor did not have a chart history of dementia, although medication history included cholinesterase inhibitors. As Montreal cognitive assessment (MoCA) done 6 and 2 years prior to death were normal, this patient was considered to be PD without dementia. When clinical information was deemed to be inadequate for the above clinical features, the sample was removed from the analysis. For DGE analysis, limma was performed as described above, using PDD, PD, and Control as groups and using “0 + group + age” as the design for limma. The contrast between PDD - PD was evaluated for each brain region. Significant genes were defined as those with an FDR <[0.05] and log_2_FC >[0.1] or <[−0.1]. GSVA was performed as described above.

#### Differential gene expression—cognitive impairment (blood)

For comparisons of cognitive impairment among the PPMI subjects, subjects with MoCa ≥26 were considered to be of normal cognition, while subjects with MoCa <26 were considered to have cognitive impairment. Only subjects with MoCa ≥26 were included among healthy controls. Limma was performed to compare PD patients with and without cognitive impairment, with age, gender, quality, and batch also included in the limma model. For the drug-naive cohort, patients who were on drugs for cognitive impairment (Galantamine, Memantine, Donepezil or Rivastigmine) were removed from the analysis, and the most recent sample when patients were drug-naive were taken for comparison.

#### Differential gene expression—dyskinesia (brain)

Patients were categorized as having levodopa-induced dyskinesia based on the mention of the term upon chart review. Samples were grouped as PD-dyskinesia or PD-no dyskinesia and control. Limma was performed as described above, using PD-dyskinesia, PD-no dyskinesia, and control as groups and using “0 + group + age” as the design for limma. For comparisons, PD-dyskinesia - PD-no dyskinesia was evaluated for each brain region. Top genes were defined as those with an FDR <[0.1] and log_2_FC >[0.1] or <[−0.1]. GSVA was performed as described above.

#### Differential gene expression—dyskinesia (blood)

Dyskinesia scores were extracted from the MDS-UPDRS 4.1 question (“Time spent with dyskinesia”) from PPMI subjects. About 201 PD subjects had a score of 0 (never had dyskinesia) and 213 subjects had a score greater than 0 (at the visit where the sample was drawn or in the past medical history). Limma was performed as described above, using PD-dyskinesia, PD-no dyskinesia, with age, gender, quality and batch included in the limma model.

#### Differential gene expression—age of onset (brain)

Age of onset was defined as the age of symptom onset (when available) or the age of diagnosis for each patient, whichever was the earliest information available. Samples were grouped into Control, LOPD (age of onset >55), or EOPD (age of onset ≤55). Limma was performed as described above, accounting for death age in the model and testing EOPD - Control and LOPD - Control for each brain region. GSVA was performed as described above.

#### Differential gene expression—age of onset (blood)

EOPD and LOPD was defined among the PPMI patients as described for the brain. Limma was performed to compare PD and control, using gender, quality, and batch in the limma model.

#### Pathway analysis—disease duration (spline interpolation in brain)

Disease duration was defined as the difference between the age of symptom onset or diagnosis (as defined above) and the age of death. Spline curves were generated from the disease duration using the ns function from the splines R package (CRAN), using 4 degrees of freedom. To allow for the determination of genes and GO terms with non-linear trends in the two brain regions, “X + region + region:X” was used as the design matrix, with X being the disease duration splines. For the determination of genes with significant trends, voomWithQualityweights was used, followed by lmFit and eBayes, as described above. To determine significance, all coefficients corresponding to the region of interest were included in the topTable function, which generates an F-statistic and *p* value. For pathway analysis, the GSVA package was used to create GSVA scores. Array weights were included in the lmFit using the arrayWeights function from limma, and the resulting object was subject to eBayes and decideTests as described above.

### Statistics and reproducibility

For differential gene expression analysis between controls and PD, all 35 PD and 40 control specimens (caudate and putamen) obtained from NIH NeuroBiobank were included and processed for bulk RNA-seq as independent biological replicates. PD caudate (*n* = 35) samples were further categorized as PD dementia (PDD), PD without dementia (PD), PD with and without dyskinesia, LOPD, and EOPD according to clinical records and described in the methods section and supplementary data files. Where clinical charts had inadequate information, samples were excluded from the analysis. For clinical variables (dementia, dyskinesia, age of onset), all available samples that met the criteria were used for analysis. For differential gene expression analysis between controls and PD in the PPMI blood, all 195 controls and 479 samples with the most likely primary diagnosis of PD that were available at the time of download in August 2019 from PPMI were included. RNA sequencing from only the latest available visit was included. Subjects with a primary diagnosis of PD were further divided into having cognitive impairment if MoCA <26 or with normal cognition if MoCA ≥26. Where clinical information did not document MoCA at the corresponding site visit, samples were excluded from the analysis. Subjects were grouped into earlier onset (EOPD) if onset ≤55 and later onset (LOPD) if onset >55 without exclusions. For all analysis including genetic carrier comparison, sample information corresponding to the visit that did not have age, sex, plate, and batch were excluded from the analysis. For PDBP, as power analysis determined that a minimum of 142 samples per group were required to detect a 1.2-fold change in RNA with a coefficient of variation = 0.50 and 20X coverage, differential RNA-seq was performed with a random sampling of 200 control and PD subjects. Statistical differences between groups were determined by two-sided Wilcoxon, Kolmogorov–Smirnov, Fisher test, and moderated *t*-tests with limma and FDRs were determined by multiple test corrections using the Benjamini–Hochberg (“BH”) method as described in the relevant sections in the Figure legends, methods and supplementary data. The investigators were not blinded to allocation during experiments and outcome assessment.

### Reporting summary

Further information on research design is available in the Nature Portfolio Reporting Summary linked to this article.

## Supplementary information

Supplementary Information

Peer Review File

Description of Additional Supplementary Files

Supplementary Data 1–49

Reporting Summary

## Data Availability

All demographic information and metadata used in this study for brain tissue analysis are available in the supplementary data files. The resulting data from all relevant limma, GSEA, and GSVA analyses for brain and blood are also included in the supplementary data files. Raw data generated from this study and gene expression values for brain RNA-seq data used in this study have been deposited in the GEO database under the accession code GSE205450 (link). Raw data and individual-level metadata used for blood transcriptome analysis in this study are available for downloading from PPMI (https://www.ppmi-info.org/access-data-specimens/download-data) through Laboratory of Neuro Imaging (LONI) Image Data Archive (IDA). For PDBP, data are available for downloading from AMP PD (https://www.amp-pd.org). To access individual-level data, users need to complete a data use agreement and submit an online application. The mass spectrometry proteomics data have been deposited to the ProteomeXchange Consortium via the PRIDE^80^ partner repository with the dataset identifier PXD042154 (link). Source data are provided with this paper.

## Source data

Source Data

## Code availability

Custom codes used in this study are available on GitHub and can be accessed at https://github.com/carynhale/PD_RNAseq.

## References

[1] Poewe W (2017). Parkinson disease. Nat. Rev. Dis. Prim..

[2] de Lau LML, Breteler MMB (2006). Epidemiology of Parkinson’s disease. Lancet Neurol..

[3] Dauer, W. & Przedborski, S. Parkinson’s disease: mechanisms and models. *Neuron*. **39**, 889–909 (2003). 10.1016/s0896-6273(03)00568-3 12971891

[4] Selikhova M (2009). A clinico-pathological study of subtypes in Parkinson’s disease. Brain.

[5] Aarsland D, Andersen K, Larsen JP, Lolk A, Kragh-Sørensen P (2003). Prevalence and characteristics of dementia in Parkinson disease: an 8-year prospective study. Arch. Neurol..

[6] Schrag A, Schott JM (2006). Epidemiological, clinical, and genetic characteristics of early-onset parkinsonism. Lancet Neurol..

[7] Provost J-S, Hanganu A, Monchi O (2015). Neuroimaging studies of the striatum in cognition Part I: healthy individuals. Front. Syst. Neurosci..

[8] Grahn JA, Parkinson JA, Owen AM (2008). The cognitive functions of the caudate nucleus. Prog. Neurobiol..

[9] Holthoff-Detto VA (1997). Functional effects of striatal dysfunction in Parkinson disease. Arch. Neurol..

[10] Wright N (2020). Elevated caudate connectivity in cognitively normal Parkinson’s disease patients. Sci. Rep..

[11] Arnaldi D (2012). What predicts cognitive decline in de novo Parkinson’s disease?. Neurobiol. Aging.

[12] Rinne JO (2000). Cognitive impairment and the brain dopaminergic system in Parkinson disease:[18F] fluorodopa positron emission tomographic study. Arch. Neurol..

[13] Roussakis A, Towey D, Gennaro M, Lao-Kaim NP, Piccini P (2019). Parkinson’s disease dyskinesias possibly relate to greater dopamine transporter losses in the putamen over time. J. Neurol. Exp. Neurosci..

[14] Herz DM (2015). Abnormal dopaminergic modulation of striato-cortical networks underlies levodopa-induced dyskinesias in humans. Brain.

[15] Borrageiro G, Haylett W, Seedat S, Kuivaniemi H, Bardien S (2018). A review of genome-wide transcriptomics studies in Parkinson’s disease. Eur. J. Neurosci..

[16] Riley BE (2014). Systems-based analyses of brain regions functionally impacted in Parkinson’s disease reveals underlying causal mechanisms. PLoS ONE.

[17] Miller RM (2006). Robust dysregulation of gene expression in substantia nigra and striatum in Parkinson’s disease. Neurobiol. Dis..

[18] Xicoy H, Brouwers JF, Wieringa B, Martens GJM (2020). Explorative combined lipid and transcriptomic profiling of substantia nigra and putamen in Parkinson’s disease. Cells.

[19] Iturria-Medina Y, Khan AF, Adewale Q, Shirazi AH, Alzheimer’s Disease Neuroimaging Initiative. (2020). Blood and brain gene expression trajectories mirror neuropathology and clinical deterioration in neurodegeneration. Brain.

[20] Toden S (2020). Noninvasive characterization of Alzheimer’s disease by circulating, cell-free messenger RNA next-generation sequencing. Sci. Adv..

[21] Marek K (2011). The Parkinson progression marker initiative (PPMI). Prog. Neurobiol..

[22] Marek K (2018). The Parkinson’s progression markers initiative (PPMI) - establishing a PD biomarker cohort. Ann. Clin. Transl. Neurol..

[23] Craig DW (2021). RNA sequencing of whole blood reveals early alterations in immune cells and gene expression in Parkinson’s disease. Nat. Aging.

[24] Hänzelmann S, Castelo R, Guinney J (2013). GSVA: gene set variation analysis for microarray and RNA-seq data. BMC Bioinformatics.

[25] Shen W, Zhai S, Surmeier DJ (2022). Striatal synaptic adaptations in Parkinson’s disease. Neurobiol. Dis..

[26] Berthet A (2014). Loss of mitochondrial fission depletes axonal mitochondria in midbrain dopamine neurons. J. Neurosci..

[27] Fanning S, Selkoe D, Dettmer U (2020). Parkinson’s disease: proteinopathy or lipidopathy?. npj Parkinson’s Dis..

[28] Shih R-H, Wang C-Y, Yang C-M (2015). NF-kappaB signaling pathways in neurological inflammation: a mini review. Front. Mol. Neurosci..

[29] Kish SJ (2008). Preferential loss of serotonin markers in caudate versus putamen in Parkinson’s disease. Brain.

[30] Velayati A, Yu WH, Sidransky E (2010). The role of glucocerebrosidase mutations in Parkinson disease and Lewy body disorders. Curr. Neurol. Neurosci. Rep..

[31] Tomlinson CL (2010). Systematic review of levodopa dose equivalency reporting in Parkinson’s disease. Mov. Disord..

[32] Singleton AB (2003). alpha-Synuclein locus triplication causes Parkinson’s disease. Science.

[33] Locascio JJ (2015). Association between α-synuclein blood transcripts and early, neuroimaging-supported Parkinson’s disease. Brain.

[34] Kato H, Lu Q, Rapaport D, Kozjak-Pavlovic V (2013). Tom70 is essential for PINK1 import into mitochondria. PLoS ONE.

[35] Goetz, C. G. et al. Movement disorder society sponsored unified Parkinson’s disease rating scale revision. *PsycTESTS Dataset* (2016).

[36] Goetz CG (2004). Movement disorder society task force report on the Hoehn and Yahr staging scale: status and recommendations. Mov. Disord..

[37] Rao S, Kay Y, Herring BE (2019). Tiam1 is critical for glutamatergic synapse structure and function in the hippocampus. J. Neurosci..

[38] Cheng J (2021). The Rac-GEF Tiam1 promotes dendrite and synapse stabilization of dentate granule cells and restricts hippocampal-dependent memory functions. J. Neurosci..

[39] Pantaleo E (2022). A machine learning approach to Parkinson’s disease blood transcriptomics. Genes.

[40] Pasquini J (2019). Clinical implications of early caudate dysfunction in Parkinson’s disease. J. Neurol. Neurosurg. Psychiatry.

[41] Lewis SJG, Dove A, Robbins TW, Barker RA, Owen AM (2003). Cognitive impairments in early Parkinson’s disease are accompanied by reductions in activity in frontostriatal neural circuitry. J. Neurosci..

[42] Sawamoto N (2007). Cognitive slowing in Parkinson disease is accompanied by hypofunctioning of the striatum. Neurology.

[43] Ohlin KE (2011). Vascular endothelial growth factor is upregulated by L-dopa in the parkinsonian brain: implications for the development of dyskinesia. Brain.

[44] Westin JE (2006). Endothelial proliferation and increased blood-brain barrier permeability in the basal ganglia in a rat model of 3,4-dihydroxyphenyl-L-alanine-induced dyskinesia. J. Neurosci..

[45] Lerner RP (2017). Levodopa-induced abnormal involuntary movements correlate with altered permeability of the blood-brain-barrier in the basal ganglia. Sci. Rep..

[46] Hirano S (2008). Dissociation of metabolic and neurovascular responses to levodopa in the treatment of Parkinson’s disease. J. Neurosci..

[47] Wickremaratchi MM (2011). The motor phenotype of Parkinson’s disease in relation to age at onset. Mov. Disord..

[48] Pagano G, Ferrara N, Brooks DJ, Pavese N (2016). Age at onset and Parkinson disease phenotype. Neurology.

[49] Alves G, Wentzel-Larsen T, Aarsland D, Larsen JP (2005). Progression of motor impairment and disability in Parkinson disease: a population-based study. Neurology.

[50] Butterfield PG, Valanis BG, Spencer PS, Lindeman CA, Nutt JG (1993). Environmental antecedents of young-onset Parkinson’s disease. Neurology.

[51] Schrag A, Hovris A, Morley D, Quinn N, Jahanshahi M (2003). Young- versus older-onset Parkinson’s disease: impact of disease and psychosocial consequences. Mov. Disord..

[52] Kordower JH (2013). Disease duration and the integrity of the nigrostriatal system in Parkinson’s disease. Brain.

[53] Skrapits K (2021). The cryptic gonadotropin-releasing hormone neuronal system of human basal ganglia. Elife.

[54] Brotchie J, Fitzer-Attas C (2009). Mechanisms compensating for dopamine loss in early Parkinson disease. Neurology.

[55] Park CS, Rehrauer H, Mansuy IM (2013). Genome-wide analysis of H4K5 acetylation associated with fear memory in mice. BMC Genomics.

[56] Jalil SJ, Sacktor TC, Shouval HZ (2015). Atypical PKCs in memory maintenance: the roles of feedback and redundancy. Learn. Mem..

[57] Shema R, Sacktor TC, Dudai Y (2007). Rapid erasure of long-term memory associations in the cortex by an inhibitor of PKMζ. Science.

[58] Gatt AP (2016). Dementia in Parkinson’s disease is associated with enhanced mitochondrial complex I deficiency. Mov. Disord..

[59] Bryois J (2020). Genetic identification of cell types underlying brain complex traits yields insights into the etiology of Parkinson’s disease. Nat. Genet..

[60] Krezel W (1998). Impaired locomotion and dopamine signaling in retinoid receptor mutant mice. Science.

[61] Toresson H, Mata de Urquiza A, Fagerström C, Perlmann T, Campbell K (1999). Retinoids are produced by glia in the lateral ganglionic eminence and regulate striatal neuron differentiation. Development.

[62] Piechota M (2016). Is senescence-associated β-galactosidase a marker of neuronal senescence?. Oncotarget.

[63] Jurk D (2012). Postmitotic neurons develop a p21-dependent senescence-like phenotype driven by a DNA damage response. Aging Cell.

[64] Chinta SJ (2018). Cellular senescence is induced by the environmental neurotoxin paraquat and contributes to neuropathology linked to Parkinson’s disease. Cell Rep..

[65] Schnöder L (2016). Deficiency of neuronal p38α MAPK attenuates amyloid pathology in Alzheimer disease mouse and cell models through facilitating lysosomal degradation of BACE1. J. Biol. Chem..

[66] Taylor DM (2013). MAP kinase phosphatase 1 (MKP-1/DUSP1) is neuroprotective in Huntington’s disease via additive effects of JNK and p38 inhibition. J. Neurosci..

[67] Bussian TJ (2018). Clearance of senescent glial cells prevents tau-dependent pathology and cognitive decline. Nature.

[68] Zhang P (2019). Senolytic therapy alleviates Aβ-associated oligodendrocyte progenitor cell senescence and cognitive deficits in an Alzheimer’s disease model. Nat. Neurosci..

[69] Gonzales MM (2021). A geroscience motivated approach to treat Alzheimer’s disease: senolytics move to clinical trials. Mech. Ageing Dev..

[70] Vorperian SK, Moufarrej MN, Quake SR, Tabula Sapiens Consortium (2022). Cell types of origin of the cell-free transcriptome. Nat. Biotechnol..

[71] Ding S-L (2017). Comprehensive cellular-resolution atlas of the adult human brain. J. Comp. Neurol..

[72] Roberts, G. W., Leigh, P. N. & Weinberger, D. R. Neuropsychiatric disorders, Ch 2 (1) (Gower Medical Publishers, 1993).

[73] Ritchie ME (2015). limma powers differential expression analyses for RNA-sequencing and microarray studies. Nucleic Acids Res..

[74] Liu R (2015). Why weight? Modelling sample and observational level variability improves power in RNA-seq analyses. Nucleic Acids Res..

[75] Korotkevich, G. et al. Fast gene set enrichment analysis. Preprint at *bioRxiv*10.1101/060012 (2016).

[76] Robinson MD, McCarthy DJ, Smyth GK (2010). edgeR: a Bioconductor package for differential expression analysis of digital gene expression data. Bioinformatics.

[77] Young MD, Wakefield MJ, Smyth GK, Oshlack A (2010). Gene ontology analysis for RNA-seq: accounting for selection bias. Genome Biol..

[78] Goksuluk D (2019). MLSeq: machine learning interface for RNA-sequencing data. Comput. Methods Prog. Biomed..

[79] Soneson C, Love MI, Robinson MD (2015). Differential analyses for RNA-seq: transcript-level estimates improve gene-level inferences. F1000Res..

[80] Perez-Riverol Y (2022). The PRIDE database resources in 2022: a hub for mass spectrometry-based proteomics evidences. Nucleic Acids Res..

