# Supplementary material for: Blood transcriptomic signatures associated with molecular changes in the brain and clinical outcomes in Parkinson’s disease
Source: Nat Commun. 2023 Jul 5;14:3956. doi: 10.1038/s41467-023-39652-6 (PMC10322907; doi:10.1038/s41467-023-39652-6)
Supplement: Supplementary file 1 — Supplementary Information [file 41467_2023_39652_MOESM1_ESM.pdf]

Supplementary Figures

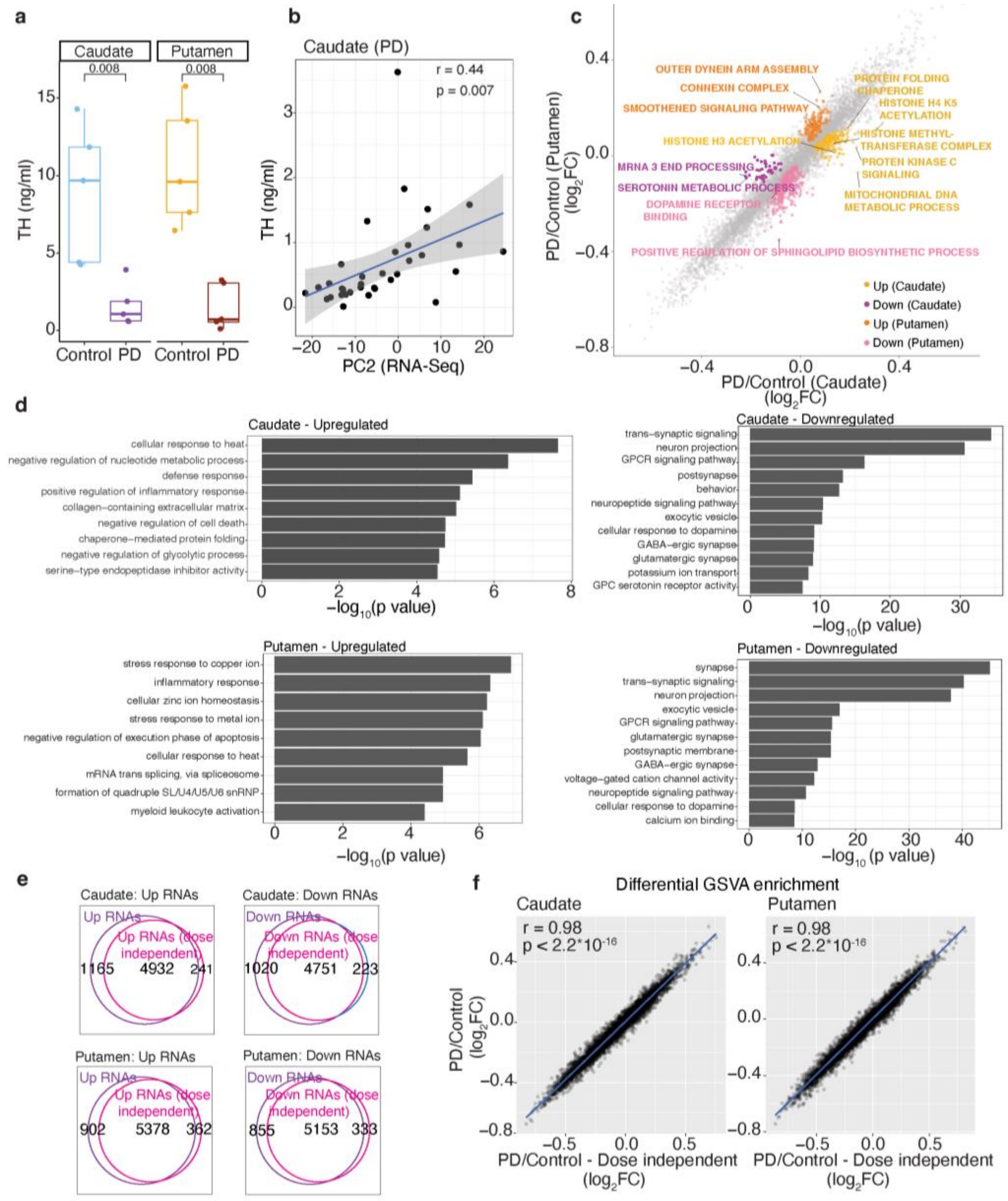

Supplementary Figure 1. Transcriptome changes in PD striatum (a) Tyrosine hydroxylase (TH) protein levels in PD caudate (n=5) and putamen (n=5) are significantly lower compared to controls (n=5). TH levels were estimated by ELISA in 120 micrograms of total protein from respective regions. p values were obtained from unpaired two-tailed Student's t-test. Box plots show lower and upper hinges corresponding to the first and third quartiles (representing 25th and 75th percentile respectively). Whiskers extend from the hinge to 1.5 * inter-quartile range. Center line indicates median. (b) Principal component 2 scores of RNA-seq (from Figure 1b) correlate with TH levels in PD caudate (n=35). Linear regression lines are shown in blue, with 95% confidence intervals shaded in gray. Pearson's test correlation values are shown. (c) Scatter plot of changes in enrichment scores (log_2FC) for GO terms that are preferentially enriched (determined by GSVA) in PD caudate and putamen compared to respective controls are highlighted. GO terms were highlighted as preferentially enriched in caudate if FDR < 0.05 in caudate and FDR > 0.1 in putamen. Likewise, preferentially enriched GO terms in putamen had FDR < 0.05 in putamen and FDR > 0.1 in caudate. Gray dots indicate all GO terms in analysis. (d) Gene ontology analysis of highly changed RNAs (FDR < 0.05, absolute value of log_2 FC > 1) in PD caudate and putamen compared to respective controls. p values are determined by goseq, which uses the Wallenius approximation for computing over- and underrepresented GO terms. (e) Venn diagrams show overlap of differentially expressed RNAs in PD and RNAs changed independent of dopaminergic medication dose in PD. Only RNAs with absolute $log_2FC > 0.1$ and FDR < 0.05 are shown (f) Correlation plot of differentially changed pathways (log_2FC, calculated by GSVA) in PD and pathways changed in PD independent of medication dose, compared to controls. Correlation coefficient (r) and p values were determined by two-sided Pearson's correlation test. Source data are provided as a Source Data file.

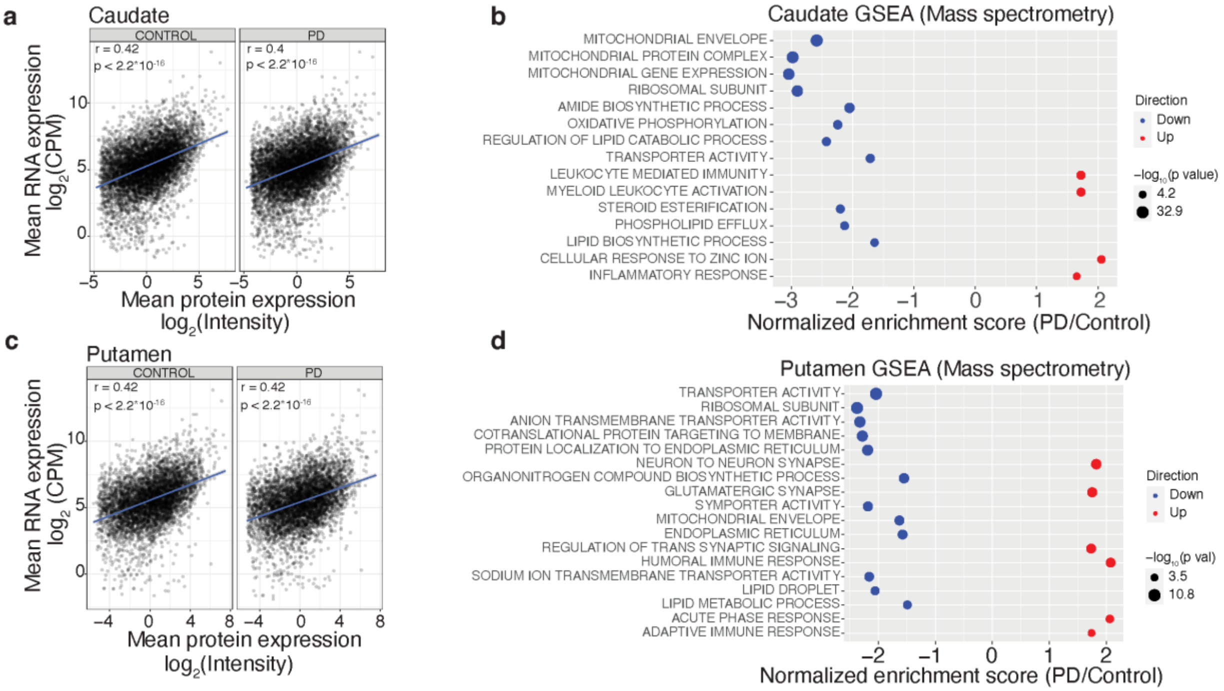

Supplementary Figure 2. Mass spectrometry in PD striatum (a) Correlation plot for mean RNA expression (RNA-seq) and protein expression (mass spectrometry) in control (RNA-seq, n=40; Mass spectrometry, n=5) and PD caudate (RNA-seq, n=35; Mass spectrometry, n=10). Statistics from two-sided Pearson correlation test are shown. (b) Gene set enrichment analysis (using fgsea with Benjamini-Hochberg (BH) adjusted p-values) shows top differentially changed pathways on mass spectrometry in PD caudate (c) Correlation plot for mean RNA and protein expression in control (RNA-seq, n=40; Mass spectrometry, n=6) and PD (RNA-seq, n=35; Mass spectrometry, n=12) putamen. Statistics from two-sided Pearson correlation test are shown. (d) GSEA for top differentially changed pathways (using fgsea with BH-adjusted p-values) on mass spectrometry in putamen.

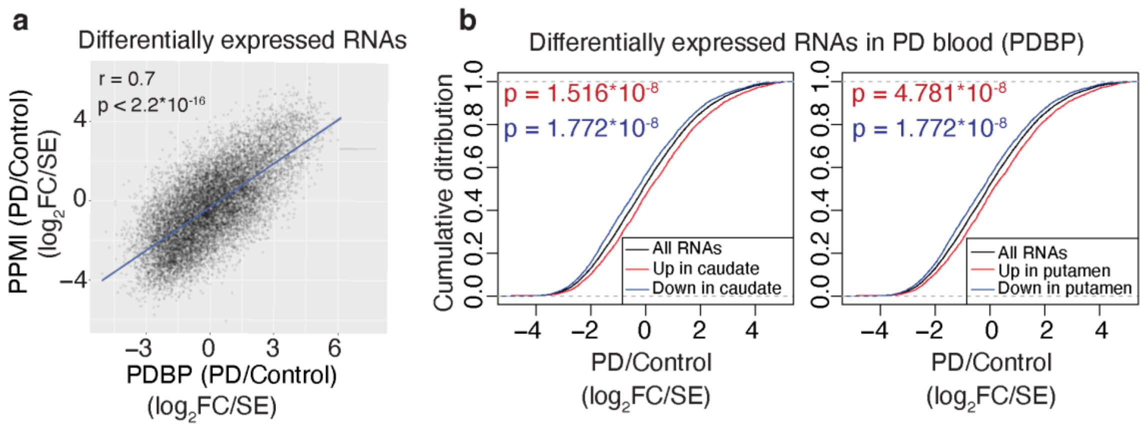

Supplementary Figure 3. Concordance with blood from PD subjects in PDBP cohort (a) Correlation between differentially expressed RNAs in PD subjects in the PPMI study, and a randomly sampled set of PD subjects in the PDBP study, compared to their respective controls. For PDBP, the randomly sampled cohort consisted of 200 PD and control subjects each. Correlation coefficient and p values was obtained from two-sided Pearson's correlation test. Blue line implies *y*=*x*. (b) RNAs up- and downregulated in PD striatum cumulatively show similar change in direction in the blood of a randomly sampled cohort enrolled in the PDBP study. A representative plot of six randomly sampled cohorts of controls and PD subjects (n=200 each) is shown. p values indicate results of two-sided Kolmogorov-Smirnov tests.

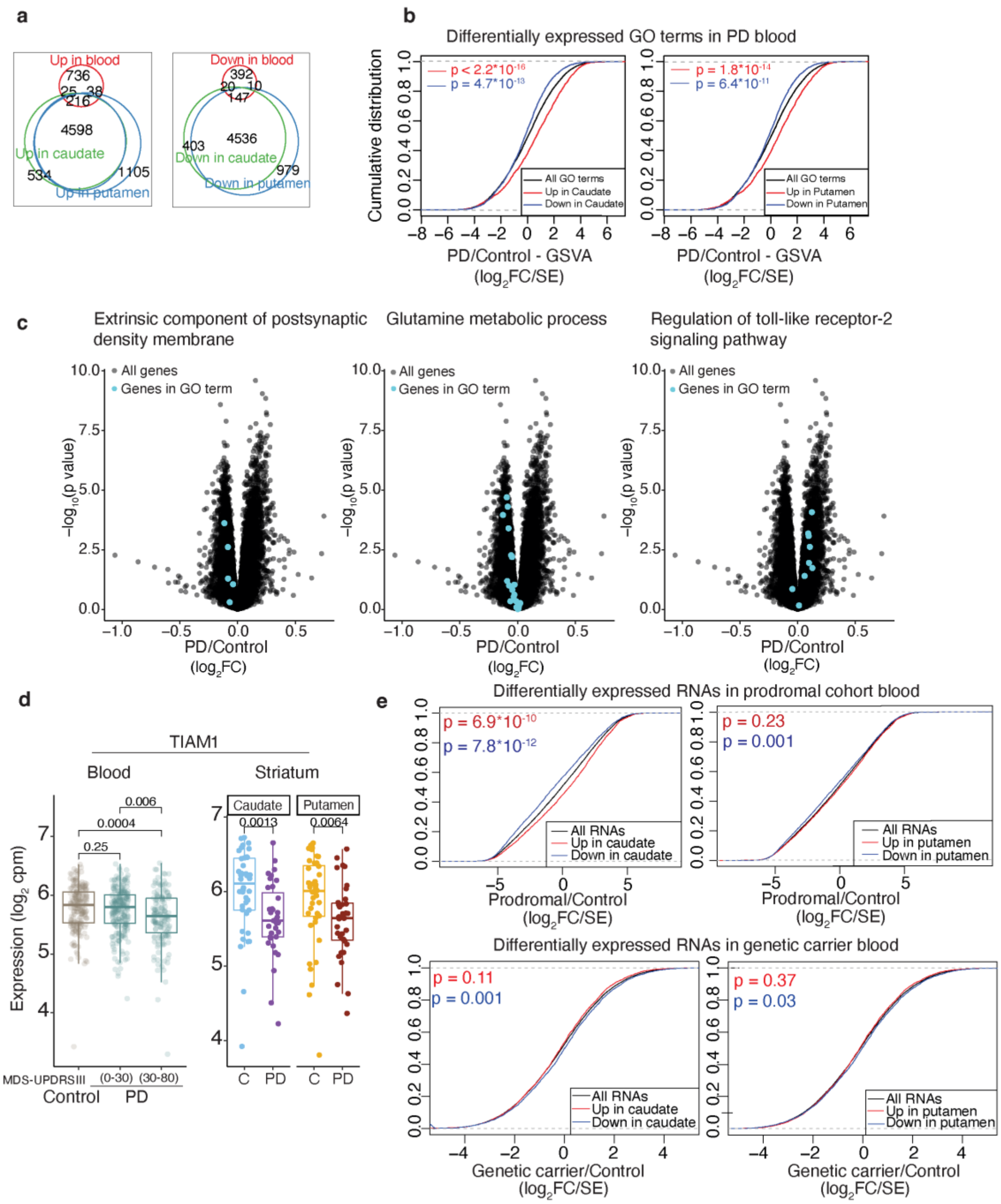

Supplementary Figure 4. Concordance between blood and brain RNA changes in PD. (a) Venn diagram showing overlap of RNAs that are significantly (FDR < 0.05) up- ($log_2FC > 0.1$) and down- ($log_2FC < -0.1$) -regulated in the caudate and putamen and blood from controls (n=195) and PD (n = 479) subjects in the PPMI cohort. (b) CDF plot of changes in GO term enrichment scores (log_2FC/SE) shows that GO terms that are significantly (FDR < 0.05) up- ($log_2FC > 0$) and down- ($log_2FC < 0$) -regulated in the PD brain as identified by GSVA are collectively up and downregulated in the blood, respectively. p values were calculated using two-sided Kolmogorov-Smirnov tests. (c) Volcano plots show that direction of change in top differentially changed GO terms (calculated by moderated t-tests and Benjamini-Hochberg correction for multiple testing with limma) in PD blood are driven by changes in most RNAs in the GO term. All RNAs are plotted in black, and RNAs within the indicated GO term are shown in blue. (d) Boxplots show that RNA expression levels ($log_2(cpm)$) of *TIAM-1*, one of the RNAs in the GO term extrinsic component of postsynaptic density membrane, is downregulated in the blood of PPMI PD subjects (UPDRS 0-30 (n=224), 30-80 (n =144), and correlates with motor progression as shown by MDS-UPDRSIII scores. *TIAM-1* is also downregulated in postmortem PD (n=35) caudate and putamen compared to controls (C, n = 40). Significance was determined by two-sided Wilcoxon tests. Box plots show lower and upper hinges corresponding to the

first and third quartiles (representing 25th and 75th percentile respectively). Whiskers extend from the hinge to 1.5 * inter-quartile range. Center line indicates median. (e) CDF plots show that differentially changed RNAs in PD striatum (caudate) show concordant changes in the blood of prodromal subjects (n=60)[23] but not in genetic carriers (n=247) who are otherwise healthy. p values were obtained from two-sided Kolmogorov-Smirnov tests. Source data are provided as a Source Data file.

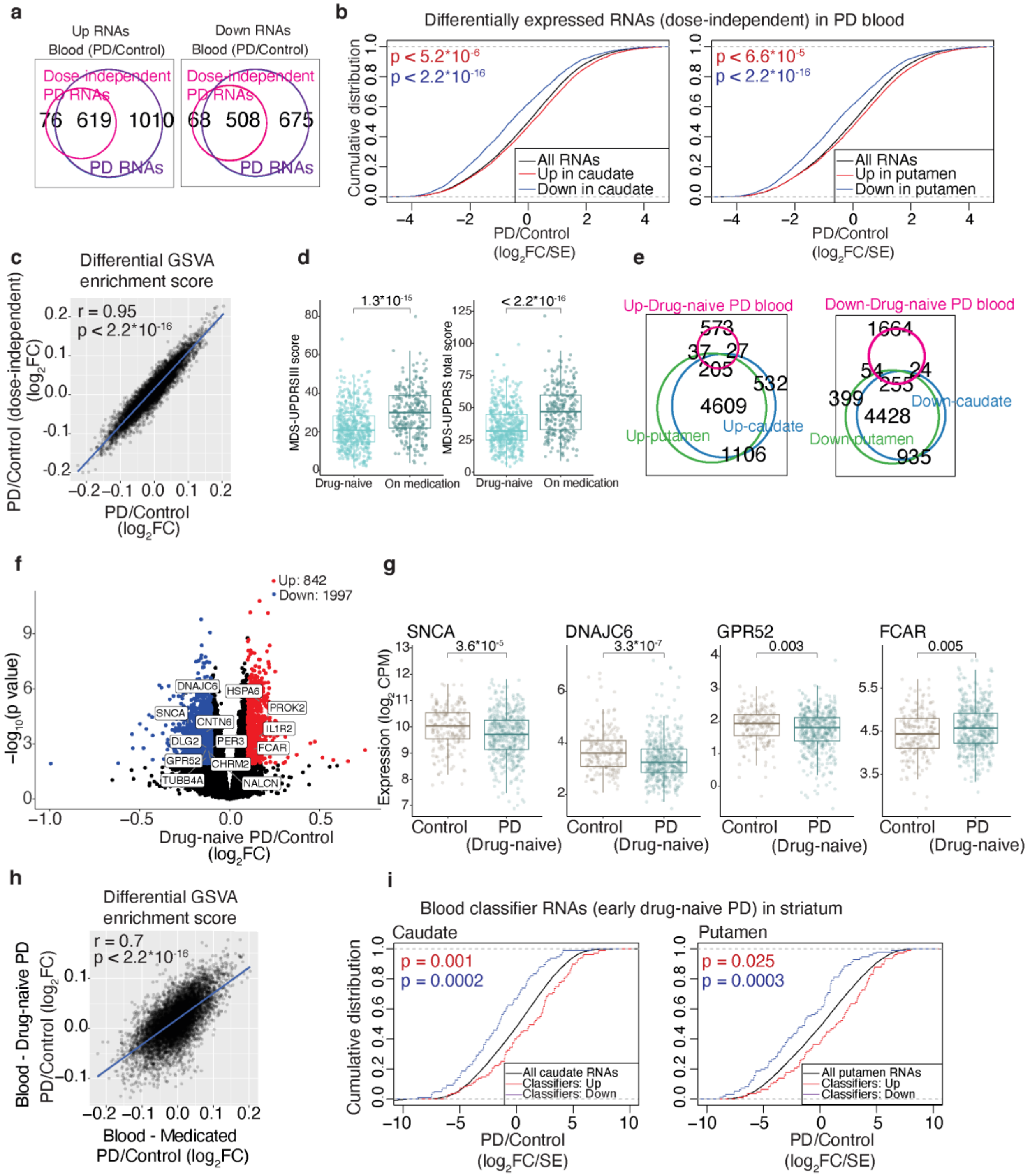

Supplementary Figure 5. Concordant RNA changes seen in PD blood and brain are independent of medication effect. (a) Venn diagrams show overlap of differentially expressed RNAs in PD and RNAs changed independent of medication dose, among PPMI subjects on dopaminergic medications when compared to controls. (b) Cumulative distribution plot of gene expression changes in the blood (log_2FC/SE) shows that differentially (FDR < 0.05, absolute value of $log_2FC > 0.1$ or $log_2FC < -0.1$) expressed RNAs in PD blood after accounting for medication dose (LEDD) show concordant changes with RNAs in the PD striatum. p values were obtained from two-sided Kolmogorov-Smirnov tests. (c) Changes in pathway enrichment (log_2FC) are plotted both before and after accounting for medication dose. Blue line indicates $y=x$. Statistics were obtained from two-sided Pearson's correlation test. (d) MDS-UPDRS total and motor (MDS-UPDRS III) scores are plotted in drug-naïve (n=463) and PD subjects on medication (n=357) in the PPMI cohort. Significance was obtained from two-sided Wilcoxon tests. (e) Overlap of blood transcriptome data obtained from drug-

naive PD subjects (n=463), with postmortem striatum RNA changes in the PD caudate and putamen. (f) Volcano plot shows significantly (FDR < 0.05) changed (absolute value of $log_2FC > 0.1$) RNAs in drug-naive PD blood compared to controls. Select brain RNAs changed in the same direction are highlighted. p values are determined by moderated t-tests with limma and multiple test correction using the BH method using log_2cpm values as input. (g) Expression plots for RNAs changed in PD striatum such as *SNCA*, the PD gene *DNAJC6*, *GPR52* and *FCAR* show similar changes in blood in drug-naive PD subjects (n=463) compared to controls (n=195). Significance was obtained from two-sided Wilcoxon tests. (h) Correlation plot comparing changes between enrichment scores (log_2FC) in the blood of PD subjects on dopaminergic medications and drug-naive subjects when compared to controls. Statistics were obtained from two-sided Pearson's correlation test. (i) Classifier RNAs identified in drug-naive early PD[39] show similar changes in postmortem PD striatum. Significance was obtained from two-sided Kolmogorov-Smirnov tests. Box plots show lower and upper hinges corresponding to the first and third quartiles (representing 25th and 75th percentile respectively). Whiskers extend from the hinge to 1.5 * inter-quartile range. Center line indicates median. Source data are provided as a Source Data file.

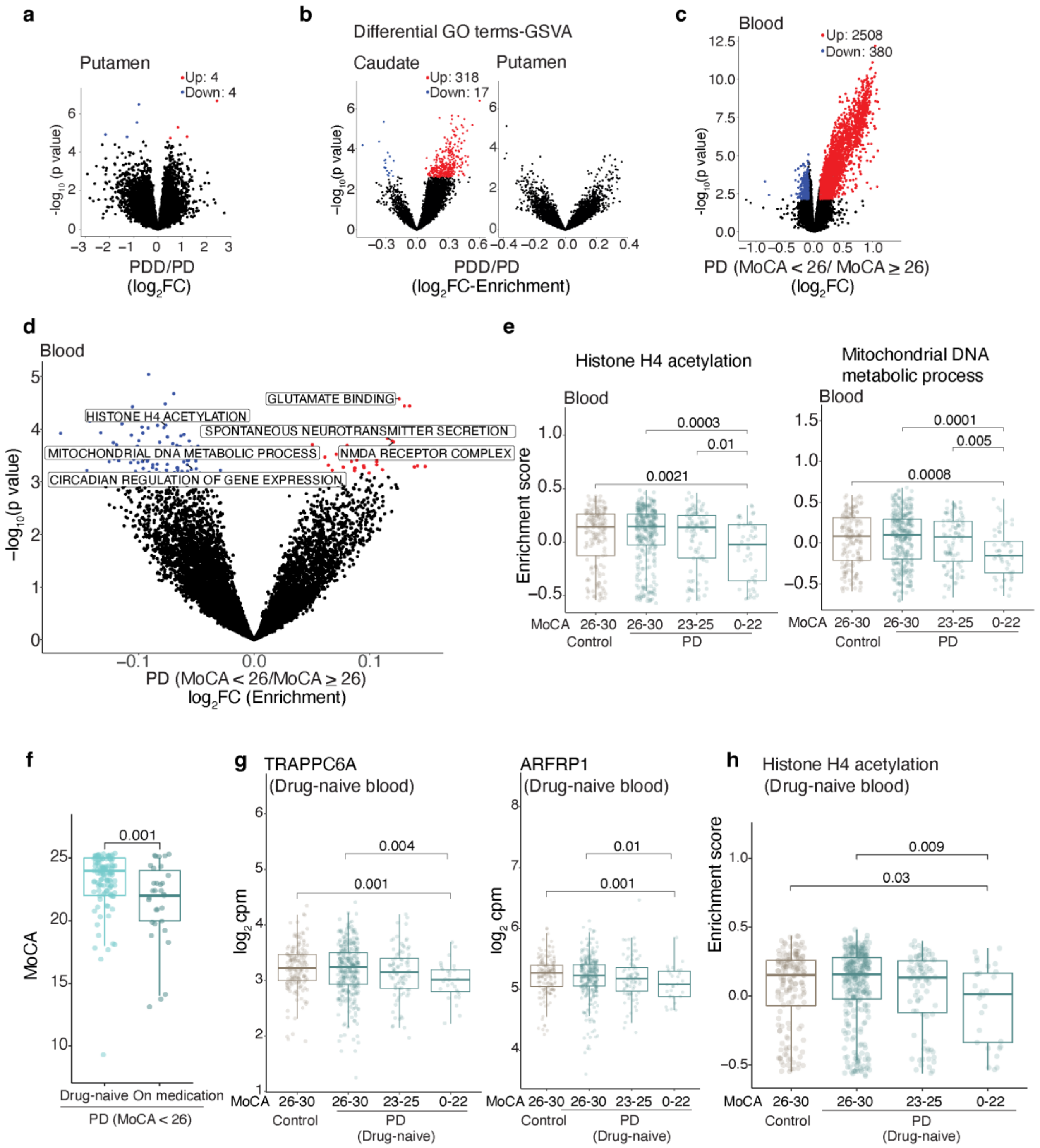

Supplementary Figure 6. Transcriptome patterns in PDD caudate are reflected in the blood of PD subjects with cognitive impairment, including those who were drug-naive. (a) Volcano plot shows few differentially expressed RNAs in PDD putamen (n=16) compared to PD without dementia (PD) (n=11). Significantly (FDR < .05) changed (absolute value of $log_2FC > 0.1$) RNAs are colored according to direction. p values are determined by moderated t-tests with limma and multiple test correction using the BH method using log_2cpm values as input. (b) GSVA identified significantly changed pathways in PDD caudate but not putamen. Pathways with FDR < 0.05 are colored according to direction. p values are determined by moderated t-tests with limma and multiple test correction using the BH method using GSVA scores (calculated by the GSVA package) values as input. (c) Volcano plot shows differentially expressed RNAs in PD blood of subjects with abnormal cognition (MoCA < 26, n=144) compared to those with normal MoCA scores (n=332). Only significantly (FDR< 0.05) changed (absolute value of $log_2FC > 0.1$) RNAs are colored according to direction. p values are determined by moderated t-tests with limma and multiple test correction using the BH method using log_2cpm

values as input (d) Differential enrichment of GO terms based on GSVA in the blood of PPMI PD subjects with abnormal MoCA (n=144) compared to subjects with normal MoCA scores (n=332). Only differentially changed GO terms with FDR < 0.05 and $log_2FC > 0$ and $log_2FC < 0$ are colored according to direction. Select top GO terms are labeled. p values are determined by moderated t-tests with limma and multiple test correction using the BH method using GSVA scores (calculated by the GSVA package) values as input. (e) Enrichment scores of top GO terms significantly changed in PDD caudate change with cognitive impairment (as determined by MoCA scores) in the blood of PPMI subjects. Controls (n=161), PD (MoCA 26-30, n=332, MoCA 23-25, n=96, MoCA 0-22, n=48). (f) Among PPMI subjects with abnormal MoCA scores (n=144), patients when they were drug-naive for dementia medications (n=112) had significantly better cognitive performance than those on medications for dementia (n=32). (g) PPMI patients naive for dementia medications were grouped according to MoCA scores and expression levels of RNAs significantly changed in the PDD caudate are plotted in the blood for these groups. Controls (n =160), PD (MoCA 26-30, n=319, MoCA 23-25, n=83, MoCA 0-22, n=29) (h) Enrichment scores of top pathways changed in PDD caudate were also changed in the drug-naive blood. Controls (n =160), PD (MoCA 26-30, n=319, MoCA 24-25, n=83, MoCA 0-22, n=29). Only significant comparisons as determined by two-sided Wilcoxon tests are shown. Box plots show lower and upper hinges corresponding to the first and third quartiles (representing 25th and 75th percentile respectively). Whiskers extend from the hinge to 1.5 * inter-quartile range. Center line indicates median. Source data are provided as a Source Data file.

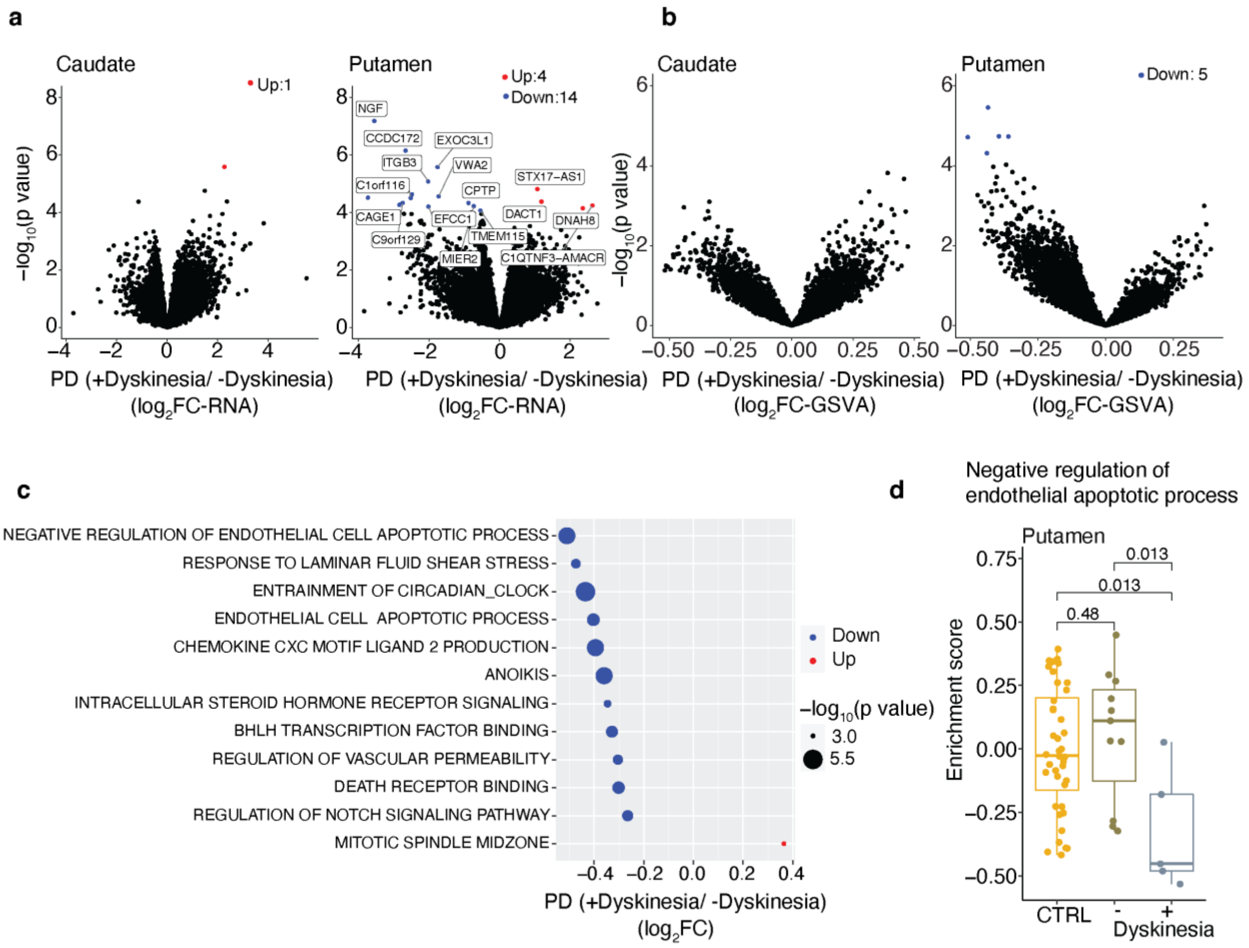

Supplementary Figure 7. Putamen is preferentially affected in PD levodopa-induced dyskinesia. (a) Volcano plot shows differentially expressed RNAs in PD dyskinesia (n=5) putamen compared to PD without dyskinesia (n=11). Only RNAs with FDR < 0.1 and absolute value of log_2FC > 0.1 are colored according to direction. Similar analysis in the caudate show few differentially expressed RNAs between those with and without dyskinesia (b) Volcano plot shows differentially enriched GO terms in PD dyskinesia compared to PD without dyskinesia in the putamen than in the caudate. Only GO terms that are differentially (FDR < 0.1) changed are colored according to direction. (c) Bubble plot shows top differentially regulated GO terms from GSVA in the putamen in patients with and without dyskinesia. BHLH: basic helix-loop-helix (d) Gene set enrichment scores of top differentially affected GO term plotted in controls and PD patients without and with dyskinesia shows downregulation of the gene set in PD dyskinesia putamen. Controls (n=40), PD - Dyskinesia (n=11), PD +Dyskinesia (n=5). p-values are the result of two-sided Wilcoxon test. Box plots show lower and upper hinges corresponding to the first and third quartiles (representing 25th and 75th percentile respectively). Whiskers extend from the hinge to 1.5
* inter-quartile range. Center line indicates median.

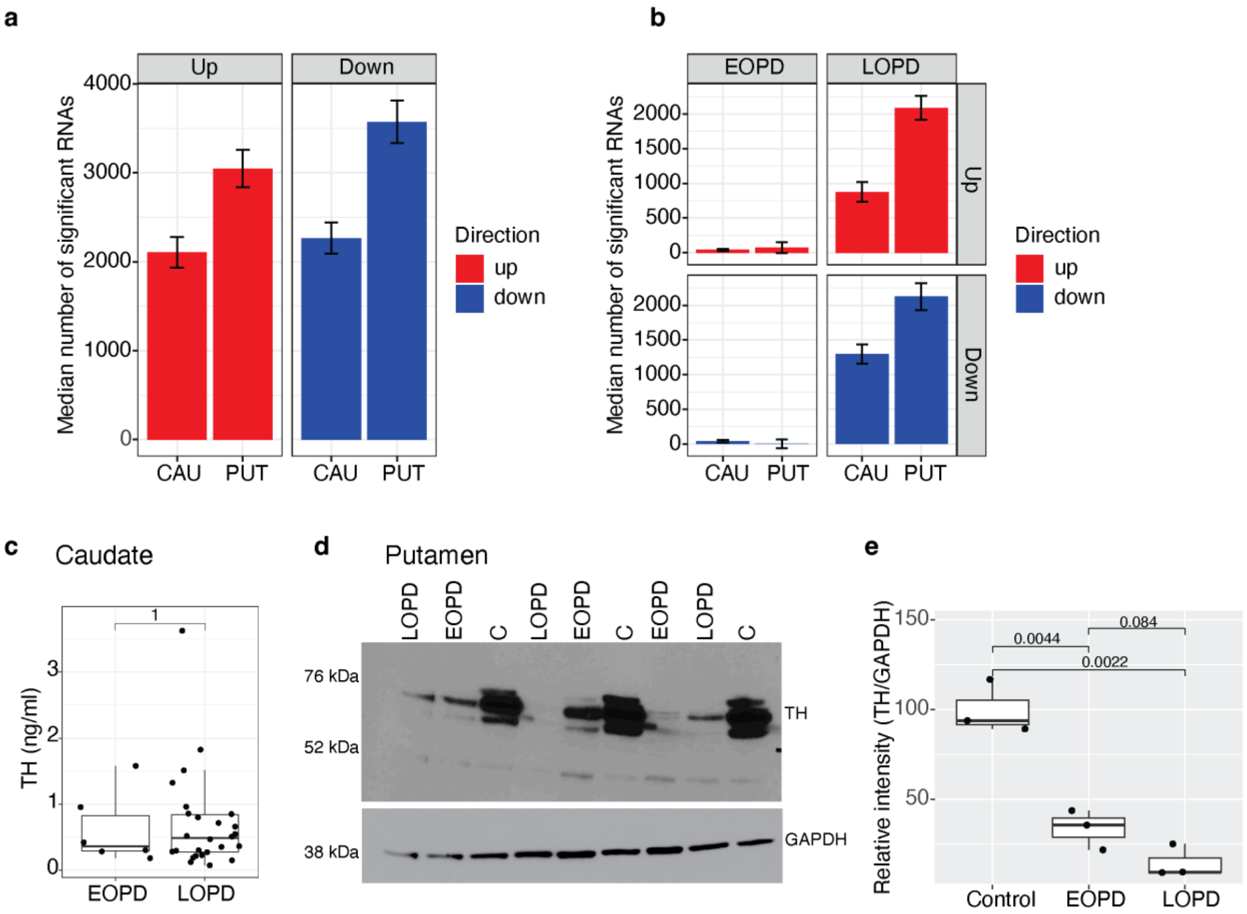

Supplementary Figure 8. LOPD and EOPD striatum are molecularly distinct with comparable loss of dopamine input (a) Differential gene expression analysis was performed on 50 randomly chosen groups (n=6) of LOPD compared to all controls (n=40). Median numbers of significantly (FDR < 0.05 and absolute $log_2FC > 0.1$) changed RNAs in these analyses are shown, grouped according to the direction of change. CAU: Caudate, PUT: Putamen Error bars indicate the mean +/- the standard error (n = 50). (b) Differential gene expression analysis was performed on 50 different groups of equal number (n=6) of randomly chosen controls and LOPD, and all 6 EOPD donors. Median numbers of significantly changed RNAs from analyses are shown. Error bars indicate the mean +/- the standard error (n = 50). (c) Enzyme-linked immunosorbent assay shows comparable levels of Tyrosine hydroxylase (TH), a marker for dopamine innervation into the striatum in EOPD (n=6) and LOPD (n=25) donors in the caudate. Significance was determined by unpaired two-tailed t-tests. (d) Western blot for TH in putamen shows decreased dopamine innervation in both LOPD and EOPD compared to controls (C) (e) Quantification of relative intensity of TH (from d) is normalized to GAPDH in LOPD and EOPD compared to controls. n=3 per group. Significance was determined by unpaired two-tailed Student's t-test. Box plots show lower and upper hinges corresponding to the first and third quartiles (representing 25th and 75th percentile respectively). Whiskers extend from the hinge to 1.5 * inter-quartile range. Center line indicates median. Source data are provided as a Source Data file.

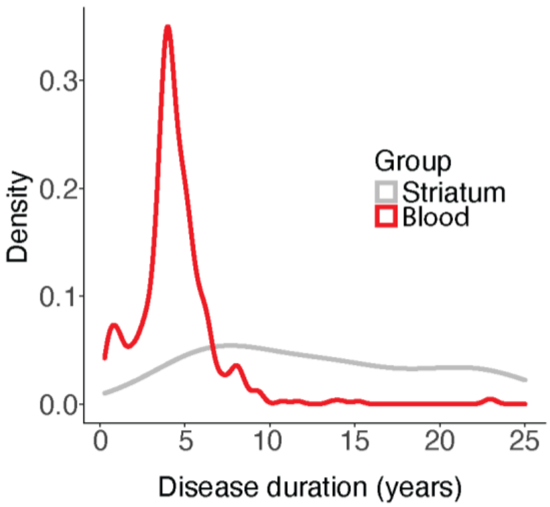

Supplementary Figure 9. Density plot shows distribution of disease duration in brain donors and subjects in the PPMI cohort.

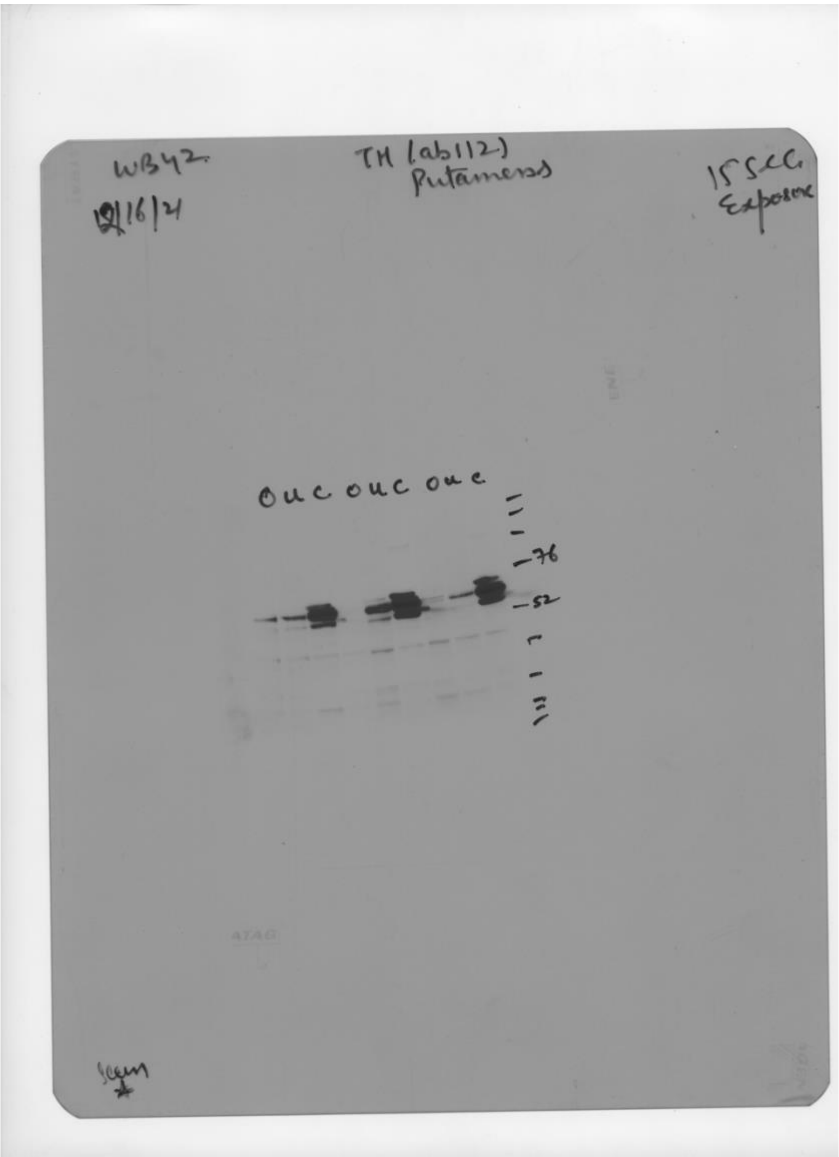

Uncropped image for Supplementary Figure 8d. Western blot with anti-TH antibody in putamen shows decreased dopamine innervation in both LOPD and EOPD compared to controls

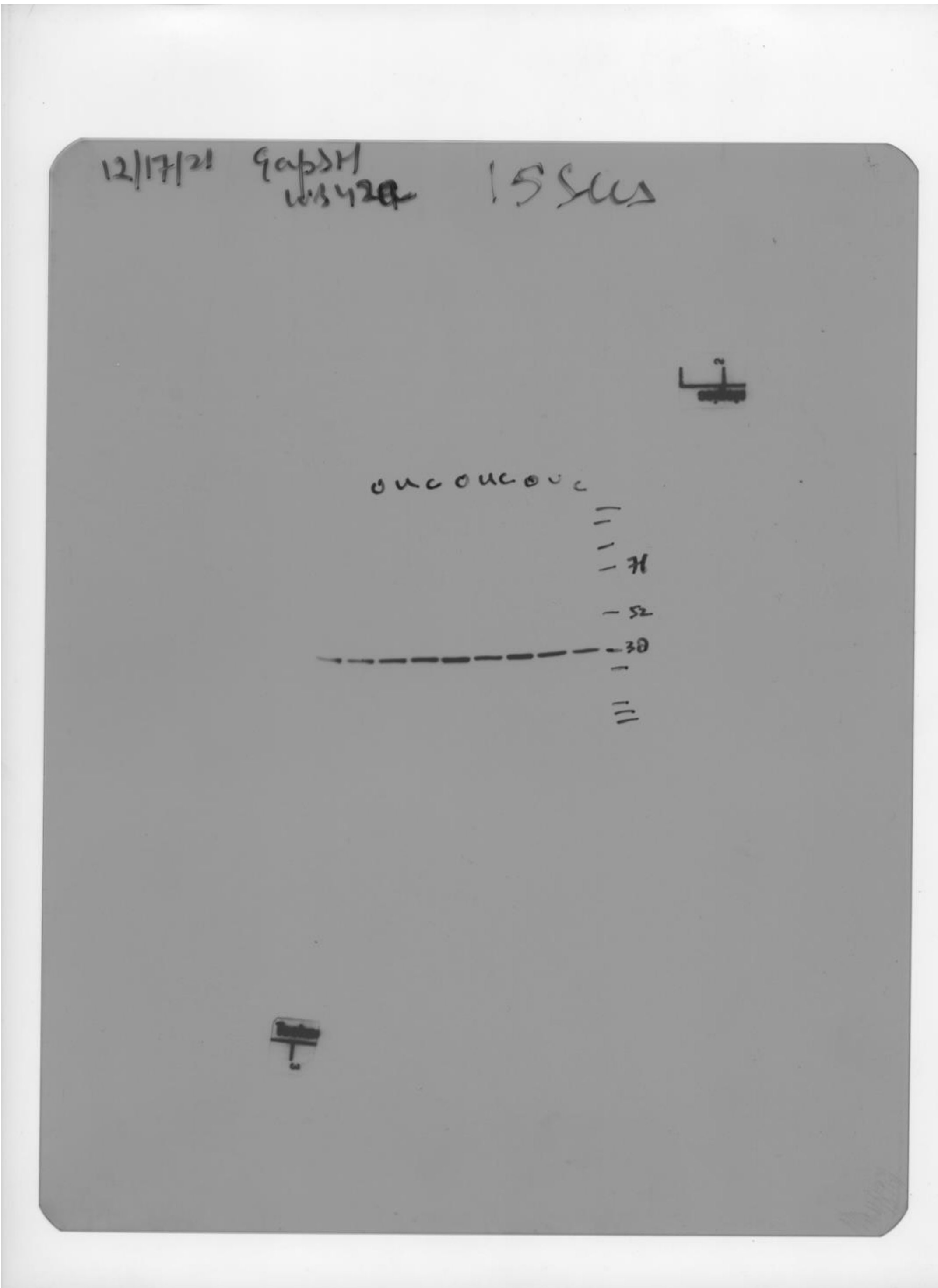

Uncropped image for Supplementary Figure 8d. Western blot with anti-GAPDH antibody as loading control
