# Supplementary material for: Blood transcriptomic signatures associated with molecular changes in the brain and clinical outcomes in Parkinson’s disease
Source: Nat Commun. 2023 Jul 5;14:3956. doi: 10.1038/s41467-023-39652-6 (PMC10322907; doi:10.1038/s41467-023-39652-6)
Supplement: Supplementary file 3 — Description of Additional Supplementary Files [file 41467_2023_39652_MOESM3_ESM.pdf]

Description of Additional Supplementary Data Files

Data S1
Metadata for all brain donors used in this study.

Data S2
Comparison of demographics for brain donors. Two-sided Wilcoxon and Fisher's tests were used for comparisons and resulting p values are given in the table.

Data S3
Protein levels (ng/ml) of Tyrosine hydroxylase detected by Enzyme-linked immunosorbent assay (ELISA) in 120 mg caudate tissue from control (n=4) and PD (n=35) donors.

Data S4
Differential gene expression analysis (measured using limma on gene counts) in putamen from PD vs control donors. p values were calculated by limma, and multiple correction was performed using the Benjamini-Hochberg method.

Data S5
Differential gene expression analysis (measured using limma) in caudate from PD vs control donors. p values were calculated by limma, and multiple correction was performed using the Benjamini-Hochberg method.

Data S6
Enrichment scores for GO terms generated from GSVA in control and PD putamen.

Data S7
Enrichment scores for GO terms generated from GSVA in control and PD caudate.

Data S8
Differentially changed GO terms (measured using limma on Enrichment scores (Data S6)) in PD vs control putamen. Enrichment scores were calculated with the GSVA R package, and limma was used to calculate p values for differential pathway expression. Multiple correction was performed using the Benjamini-Hochberg method.

Data S9
Differentially changed GO terms (measured using limma on Enrichment scores (Data S7)) in PD vs control caudate. Enrichment scores were calculated with the GSVA R package, and limma was used to calculate p values for differential pathway expression. Multiple correction was performed using the Benjamini-Hochberg method.

Data S10
List of highly (absolute ($log_2FC > 1$) and $FDR < 0.05$) differentially expressed genes (based on statistics from Data 4 and 5) in PD caudate and putamen compared to their respective controls.

Data S11
Statistics from ML-Seq analysis for identification of classifier genes in control and PD caudate. Statistics were calculated using the MLSeq R package. The confusion matrix from this analysis is shown. p values were obtained used McNemar test using default parameters from R stats package.

Data S12
Protein expression values from mass spectrometry in control and PD caudate. Significance was tested using permutation-based FDR-corrected t-tests.

Data S13
Protein expression values from mass spectrometry in control and PD putamen. Significance was tested using permutation-based FDR-corrected t-tests.

Data S14
Gene set enrichment analysis (GSEA) to identify GO terms enriched in PD vs Control protein levels in caudate (by Mass Spectrometry). p values were calculated by the fgsea R package and adjusted p values were calculated using the Benjamini-Hochberg method.

Data S15
Gene set enrichment analysis (GSEA) to identify GO terms enriched in PD vs Control protein levels in putamen (by Mass Spectrometry). p values were calculated by the fgsea R package and adjusted p values were calculated using the Benjamini-Hochberg method.

Data S16
Comparison of demographics for PPMI subjects. Two-sided Wilcoxon and Fisher's tests were used for comparisons and resulting p values are given in the table.

Data S17
Differential gene expression analysis (measured using limma) in blood from PD vs control subjects in the PPMI study. p values were calculated by limma, and multiple correction was performed using the Benjamini-Hochberg method.

Data S18
Differential gene expression analysis (measured using limma) in blood from PD vs control subjects in the PDBP study. p values were calculated by limma, and multiple correction was performed using the Benjamini-Hochberg method.

Data S19
Differentially changed GO terms in PD vs control subjects in the PPMI study. Enrichment scores were calculated with the GSVA R package, and limma was used to calculate p values for

differential pathway expression. Multiple correction was performed using the Benjamini-Hochberg method.

Data S20
Differential gene expression analysis (measured using limma) in genetic carriers vs control subjects in the PPMI study. p values were calculated by limma, and multiple correction was performed using the Benjamini-Hochberg method.

Data S21
Limma analysis to identify genes that correlate with LEDD values in PD subjects of the PPMI study. p values were calculated by limma, and multiple correction was performed using the Benjamini - Hochberg method.

Data S22
Differential gene expression analysis (measured using limma) in blood from drug naïve PD vs control subjects in the PPMI study. p values were calculated by limma, and multiple correction was performed using the Benjamini-Hochberg method.

Data S23
Differentially changed GO terms in drug naïve PD vs control subjects in the PPMI study. Enrichment scores were calculated with the GSVA R package, and limma was used to calculate p values for differential pathway expression. Multiple correction was performed using the Benjamini-Hochberg method.

Data S24
Demographics for PD donors with and without dementia. Two-sided Wilcoxon and Fisher's tests were used for comparisons and resulting p values are given in the table.

Data S25
Differential gene expression analysis (measured using limma) in putamen of PD donors with dementia vs PD donors without dementia. p values were calculated by limma, and multiple correction was performed using the Benjamini-Hochberg method.

Data S26
Differential gene expression analysis (measured using limma) in caudate of PD donors with dementia vs PD donors without dementia. p values were calculated by limma, and multiple correction was performed using the Benjamini-Hochberg method.

Data S27
Differentially changed GO terms in caudate of PD donors with dementia vs PD donors without dementia. Enrichment scores were calculated with the GSVA R package, and limma was used to calculate p values for differential pathway expression. Multiple correction was performed using the Benjamini-Hochberg method.

Data S28
Differentially changed GO terms in putamen of PD donors with dementia vs PD donors without dementia. Enrichment scores were calculated with the GSVA R package, and limma was used to calculate p values for differential pathway expression. Multiple correction was performed using the Benjamini-Hochberg method.

Data S29
Demographics for PD subjects with and without cognitive impairment in the PPMI study. Two-sided Wilcoxon and Fisher's tests were used for comparisons and resulting p values are given in the table.

Data S30
Differential gene expression in PD subjects with cognitive impairment vs PD subjects without cognitive impairment in the PPMI study. p values were calculated by limma, and multiple correction was performed using the Benjamini-Hochberg method.

Data S31
Differentially changed GO terms in PD subjects with cognitive impairment vs PD subjects without cognitive impairment in the PPMI study. Enrichment scores were calculated with the GSVA R package, and limma was used to calculate p values for differential pathway expression. Multiple correction was performed using the Benjamini-Hochberg method.

Data S32
Demographics for PD brain donors with and without history of dyskinesia. Two-sided Wilcoxon and Fisher's tests were used for comparisons and resulting p values are given in the table.

Data S33
Differential gene expression analysis (measured using limma) in putamen of PD donors with history of dyskinesia vs PD donors without history of dyskinesia. p values were calculated by limma, and multiple correction was performed using the Benjamini-Hochberg method.

Data S34
Differential gene expression analysis (measured using limma) in caudate of PD donors with history of dyskinesia vs PD donors without history of dyskinesia. p values were calculated by limma, and multiple correction was performed using the Benjamini-Hochberg method.

Data S35
Differential gene expression analysis (measured using limma) in blood of PD subjects in the PPMI study with history of dyskinesia vs PD subjects without history of dyskinesia. p values were calculated by limma, and multiple correction was performed using the Benjamini-Hochberg method.

Data S36

Differentially changed GO terms in putamen of PD donors with dyskinesia vs PD donors without dyskinesia. Enrichment scores were calculated with the GSVA R package, and limma was used to calculate p values for differential pathway expression. Multiple correction was performed using the Benjamini-Hochberg method.

Data S37

Differentially changed GO terms in caudate of PD donors with dyskinesia vs PD donors without dyskinesia. Enrichment scores were calculated with the GSVA R package, and limma was used to calculate p values for differential pathway expression. Multiple correction was performed using the Benjamini-Hochberg method.

Data S38

Demographics for earlier-onset and later-onset PD brain donors. Two-sided Wilcoxon and Fisher's tests were used for comparisons and resulting p values are given in the table.

Data S39

Differential gene expression analysis (measured using limma) in caudate of later-onset PD versus control donors. p values were calculated by limma, and multiple correction was performed using the Benjamini-Hochberg method.

Data S40

Differential gene expression analysis (measured using limma) in putamen of later-onset PD versus control donors. p values were calculated by limma, and multiple correction was performed using the Benjamini-Hochberg method.

Data S41

Differential gene expression analysis (measured using limma) in putamen of earlier-onset PD versus control donors. p values were calculated by limma, and multiple correction was performed using the Benjamini-Hochberg method.

Data S42

Differential gene expression analysis (measured using limma) in caudate of earlier-onset PD versus control donors. p values were calculated by limma, and multiple correction was performed using the Benjamini-Hochberg method.

Data S43

Differentially changed GO terms in putamen of later-onset PD donors vs control donors. Enrichment scores were calculated with the GSVA R package, and limma was used to calculate p values for differential pathway expression. Multiple correction was performed using the Benjamini-Hochberg method.

Data S44
Differentially changed GO terms in caudate of later-onset PD donors vs control donors. Enrichment scores were calculated with the GSVA R package, and limma was used to calculate p values for differential pathway expression. Multiple correction was performed using the Benjamini-Hochberg method.

Data S45
Demographics for PD subjects with earlier and later onset PD in PPMI study. Two-sided Wilcoxon and Fisher's tests were used for comparisons and resulting p values are given in the table.

Data S46
Differential gene expression analysis (measured using limma) in blood of later-onset PD versus control subjects in the PPMI study. p values were calculated by limma, and multiple correction was performed using the Benjamini-Hochberg method.

Data S47
Differential gene expression analysis (measured using limma) in blood of earlier-onset PD versus control subjects in the PPMI study. p values were calculated by limma, and multiple correction was performed using the Benjamini-Hochberg method.

Data S48
Pathway association with disease duration using splines regression models on GO term enrichment scores (Data S7)) in caudate. Enrichment scores were calculated with the GSVA R package, and limma was used to calculate p values for differential pathway expression. Multiple correction was performed using the Benjamini Hochberg method.

Data S49
Pathway association with disease duration using splines regression models on GO term enrichment scores (Data S6)) in putamen. Enrichment scores were calculated with the GSVA R package, and limma was used to calculate p values for differential pathway expression. Multiple correction was performed using the Benjamini-Hochberg method.
