# Supplementary material for: Blood transcriptomic signatures associated with molecular changes in the brain and clinical outcomes in Parkinson’s disease
Source: Nat Commun. 2023 Jul 5;14:3956. doi: 10.1038/s41467-023-39652-6 (PMC10322907; doi:10.1038/s41467-023-39652-6)
Supplement: Supplementary file 5 — Reporting Summary [file 41467_2023_39652_MOESM5_ESM.pdf]

nature portfolio

Corresponding author(s): Robert Darnell

Last updated by author(s): 05/20/2023

Reporting Summary

Nature Portfolio wishes to improve the reproducibility of the work that we publish. This form provides structure for consistency and transparency in reporting. For further information on Nature Portfolio policies, see our Editorial Policies and the Editorial Policy Checklist.

Statistics

For all statistical analyses, confirm that the following items are present in the figure legend, table legend, main text, or Methods section.

n/a	Confirmed	
☐	☒	The exact sample size (*n*) for each experimental group/condition, given as a discrete number and unit of measurement
☐	☒	A statement on whether measurements were taken from distinct samples or whether the same sample was measured repeatedly
☐	☒	The statistical test(s) used AND whether they are one- or two-sided<br>*Only common tests should be described solely by name; describe more complex techniques in the Methods section.*
☐	☒	A description of all covariates tested
☐	☒	A description of any assumptions or corrections, such as tests of normality and adjustment for multiple comparisons
☐	☒	A full description of the statistical parameters including central tendency (e.g. means) or other basic estimates (e.g. regression coefficient) AND variation (e.g. standard deviation) or associated estimates of uncertainty (e.g. confidence intervals)
☐	☒	For null hypothesis testing, the test statistic (e.g. *F*, *t*, *r*) with confidence intervals, effect sizes, degrees of freedom and *P* value noted<br>*Give P values as exact values whenever suitable.*
☒	☐	For Bayesian analysis, information on the choice of priors and Markov chain Monte Carlo settings
☒	☐	For hierarchical and complex designs, identification of the appropriate level for tests and full reporting of outcomes
☐	☒	Estimates of effect sizes (e.g. Cohen's *d*, Pearson's *r*), indicating how they were calculated

Our web collection on statistics for biologists contains articles on many of the points above.

Software and code

Policy information about availability of computer code

Data collection	No software was used to collect data
Data analysis	All packages and software described were used according to standard protocols, using functions and parameters as described in the methods section. R packages used include limma (v3.50.0), edgeR (v3.36.0), fgsea (v1.20.0), GSVA (v1.42.0), ML-Seq (v2.12.0), goseq (v1.46.0), ggplot2 (v3.5.0), ggpubr (v0.4.0), pcaExplorer (v2.20.0), splines (v4.1.2).

For manuscripts utilizing custom algorithms or software that are central to the research but not yet described in published literature, software must be made available to editors and reviewers. We strongly encourage code deposition in a community repository (e.g. GitHub). See the Nature Portfolio guidelines for submitting code & software for further information.

Data

Policy information about availability of data

All manuscripts must include a data availability statement. This statement should provide the following information, where applicable:

- Accession codes, unique identifiers, or web links for publicly available datasets
- A description of any restrictions on data availability
- For clinical datasets or third party data, please ensure that the statement adheres to our policy

All demographic information and metadata is available for brain donors in the Supplemental Table. In addition, the resulting data from all relevant limma, GSEA and GSVA analyses are also included for both brain and blood analysis. Raw data and gene expression values for brain RNA-seq data were uploaded to GEO (GSE205450). Raw data for blood transcriptome analysis and individual level metadata used in this study are available for downloading from PPMI (https://

www.ppmi-info.org/access-data-specimens/download-data) through Laboratory of Neuro Imaging (LONI) Image Data Archive (IDA) and AMP PD (https://www.amp-pd.org) for the PDBP data. To access the data, users need to complete a data use agreement and submit an online application. The mass spectrometry proteomics data have been deposited to the ProteomeXchange Consortium via the PRIDE79 partner repository with the dataset identifier PXD042154.

Human research participants

Policy information about studies involving human research participants and Sex and Gender in Research.

Reporting on sex and gender	Brain samples were from deceased de-identified donors from biorepositories and are therefore not human research participants. In comparative analysis of controls and PD in brain and blood biospecimens (from PPMI), both males and females were included in analysis. Demographic tables in the supplemental table provide full details of the gender information used in each analysis.
Population characteristics	Full details of the population characteristics including age, gender, primary diagnosis and clinical variables (e.g: cognition, dyskinesia, age of onset, medication status, disease duration) are included in the supplemental table for each analysis in brain and blood.
Recruitment	We did not perform subject recruitment.
Ethics oversight	Research conducted is in accordance with ethical guidelines and regulations. The use of postmortem brain samples obtained from brain banks was governed by a Material Transfer Agreement (MTA), which ensured that the samples were obtained and used in accordance with legal and ethical requirements. The brain banks followed appropriate consent procedures from donors or their legal representatives for the collection and distribution of postmortem brain tissue for research use. This study also incorporated publicly accessible blood transcriptome data from de-identified individuals who provided informed consent at data collection sites. The utilization of these data followed the guidelines outlined by the data repository's Data Use Access policies. The research outline and protocol, including the use of postmortem brain samples and publicly available blood transcriptome data, was reviewed by the Chair of the Rockefeller University's Institutional Review Board (IRB).

Note that full information on the approval of the study protocol must also be provided in the manuscript.

Field-specific reporting

Please select the one below that is the best fit for your research. If you are not sure, read the appropriate sections before making your selection.

☒ Life sciences ☐ Behavioural & social sciences ☐ Ecological, evolutionary & environmental sciences

For a reference copy of the document with all sections, see nature.com/documents/nr-reporting-summary-flat.pdf

Life sciences study design

All studies must disclose on these points even when the disclosure is negative.

Sample size	For comparison of controls and PD, all available brain samples from the NIH NeuroBioBank were included in the analysis. For clinical variables (dementia, dyskinesia, age of onset), all available samples that met criteria were used for analysis. For PPMI, when criteria were met, all available samples at download were used in the analysis comparing controls and PD and clinical variables within PD. Samples where plate, age, sex or batch information were not available from PPMI were excluded from analysis. For PDBP, as power analysis determined that a minimum of 142 samples per group were required to detect a 1.2 fold change in RNA with coefficient of variation = 0.50 and 20X coverage, differential RNA-seq was performed with random sampling of 200 control and PD subjects.
Data exclusions	For differential gene expression analysis between controls and PD , all 35 PD and 40 control specimens (caudate and putamen) obtained from NIH Neuobiobank were included and processed for RNA-seq as independent biological replicates. PD caudate (n=35) samples were further categorized as PD dementia (PDD), PD without dementia (PD), according to clinical records and described in the methods section and supplemental table. For selection of PDD and PD without dementia a chart history of dementia was supported by medication history for dementia or scores from a cognitive test. In those considered not to have dementia, chart reviews did not mention dementia or history of taking medication for dementia. One donor did not have a chart history of dementia although medication history included cholinesterase inhibitors. As Montreal cognitive assessment (MoCA) done 6 and 2 years prior to death were normal, this patient was considered to be PD without dementia. Patients were grouped as PD-dyskinesia or PD without dyskinesia based on mention of the term upon chart review. Age of onset was defined as the age of symptom onset (when available) or the age of diagnosis for each patient, whichever was the earliest information available. Samples were grouped into Control, later age of onset or LOPD (age of onset > than 55), or EOPD (age of onset ≤ to 55). Where clinical charts had inadequate information to gather the above information, samples were excluded from analysis.<br>For differential gene expression analysis between controls and PD in the blood, all 195 controls and 479 samples with most likely primary diagnosis of PD that were available for download from PPMI were included. RNA sequencing from only the latest available visit was included. Subjects with primary diagnosis of PD were further divided into having cognitive impairment if MoCA <26 or with normal cognition if MoCA >= 26. Where clinical information did not document MoCA at the corresponding site visit, samples were excluded from analysis. Subjects were grouped into earlier onset (EOPD) if onset <=55 and later onset (LOPD) if onset >55 without exclusions. For all analysis including genetic carrier comparison, samples that did not have age, sex, plate and batch on the PPMI site were excluded from analysis.

Replication	All available samples from NIH NeuroBioBank were used for differential gene expression analysis in the brain. RNA-sequencing was done in once from independent samples (n=35, PD; n=40, Controls). Differential transcriptome analysis in the blood comparing 479 PD subjects and 195 controls were used to identify the differentially expressed RNAs detected in PD brain. Concordance in blood was replicated in randomly sampled subsets of PDBP cohort. For ELISA for Tyrosine hydroxylase from select samples, 2-3 technical replicates were used as described in methods.
Randomization	Based on neuropathological diagnosis, all available specimens from NIH NeuroBioBank were grouped into controls and PD. voomWithQualityWeights from Limma was used for normalization to account for RNA quality and age of death was used as a covariate in the analysis. For differential expression of the PPMI cohort, only RNA sequencing from the latest site visit was used for analysis. Latest site visit was also used for drug-naive cohort. Covariates for analysis included age (except for comparison of EOPD and LOPD), gender, RIN and RNA quality similar to previous analysis of the PPMI data (Craig et al., Nature Aging, 2021).
Blinding	Data collection were performed by outside vendors for blood samples and brain banks for brain samples. Brain banks considered clinical diagnosis in final interpretation of pathological findings. Data collection for blood samples were done blinded by outside vendors. Specimens for brain and blood RNA sequencing (PPMI/PDBP) were not blinded at analysis as they clearly met criteria for one arm or the other.

Reporting for specific materials, systems and methods

We require information from authors about some types of materials, experimental systems and methods used in many studies. Here, indicate whether each material, system or method listed is relevant to your study. If you are not sure if a list item applies to your research, read the appropriate section before selecting a response.

Materials & experimental systems

n/a	Involved in the study	
☐	☒	Antibodies
☒	☐	Eukaryotic cell lines
☒	☐	Palaeontology and archaeology
☒	☐	Animals and other organisms
☒	☐	Clinical data
☒	☐	Dual use research of concern

Methods

n/a	Involved in the study	
☒	☐	ChIP-seq
☒	☐	Flow cytometry
☒	☐	MRI-based neuroimaging

Antibodies

Antibodies used	anti-tyrosine hydroxylase (Abcam, AB112); anti-GAPDH (Invitrogen, AM4300); TH ELISA kit (Antibodies online, ABIN6960326)
Validation	AB112 and AM4300 antibodies have been used in immunoblots to detect respective proteins in human tissue/cell-line.<br>AB112: https://app.benchsci.com/product/Abcam/AB112/info?product_type=antibody<br>AM4300: https://www.thermofisher.com/order/genome-database/dataSheetPdf?<br>producttype=antibody&productsubtype=antibody_primary&productId=AM4300&version=225<br>ABIN6960326: https://www.antibodies-online.com/kit/6960326/Tyrosine+Hydroxylase+TH+ELISA+Kit/
